# Age‐dependent changes in response property and morphology of a thermosensory neuron and thermotaxis behavior in *Caenorhabditis elegans*

**DOI:** 10.1111/acel.13146

**Published:** 2020-04-19

**Authors:** Tzu‐Ting Huang, Hironori J. Matsuyama, Yuki Tsukada, Aakanksha Singhvi, Ru‐Ting Syu, Yun Lu, Shai Shaham, Ikue Mori, Chun‐Liang Pan

**Affiliations:** ^1^ Neuroscience Institute Graduate School of Science Nagoya University Nagoya Japan; ^2^ Group of Molecular Neurobiology Graduate School of Science Nagoya University Nagoya Japan; ^3^ Institute of Molecular Medicine College of Medicine National Taiwan University Taipei Taiwan; ^4^ Laboratory of Developmental Genetics The Rockefeller University New York NY USA; ^5^ Center of Precision Medicine College of Medicine National Taiwan University Taipei Taiwan; ^6^ Present address: Fred Hutchinson Cancer Research Center Seattle WA USA

**Keywords:** actin, aging, behavior, *Caenorhabditis elegans*, calcium, cilia, neural plasticity, sensory neuron

## Abstract

Age‐dependent cognitive and behavioral deterioration may arise from defects in different components of the nervous system, including those of neurons, synapses, glial cells, or a combination of them. We find that AFD, the primary thermosensory neuron of *Caenorhabditis elegans*, in aged animals is characterized by loss of sensory ending integrity, including reduced actin‐based microvilli abundance and aggregation of thermosensory guanylyl cyclases. At the functional level, AFD neurons in aged animals are hypersensitive to high temperatures and show sustained sensory‐evoked calcium dynamics, resulting in a prolonged operating range. At the behavioral level, senescent animals display cryophilic behaviors that remain plastic to acute temperature changes. Excessive cyclase activity of the AFD‐specific guanylyl cyclase, GCY‐8, is associated with developmental defects in AFD sensory ending and cryophilic behavior. Surprisingly, loss of the GCY‐8 cyclase domain reduces these age‐dependent morphological and behavioral changes, while a prolonged AFD operating range still exists in *gcy‐8* animals. The lack of apparent correlation between age‐dependent changes in the morphology or stimuli‐evoked response properties of primary sensory neurons and those in related behaviors highlights the importance of quantitative analyses of aging features when interpreting age‐related changes at structural and functional levels. Our work identifies aging hallmarks in AFD receptive ending, temperature‐evoked AFD responses, and experience‐based thermotaxis behavior, which serve as a foundation to further elucidate the neural basis of cognitive aging.

## Graphical abstract

Aged *Caenorhabditis elegans* shows prolonged temperature'evoked calcium transients and altered sensory ending in thermosensory neurons, along with cryophilic behaviors.

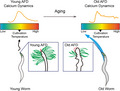

## 1 INTRODUCTION

Neural aging is characterized by progressive deterioration of structure and function of the nervous system. Impairment of synaptic structures, such as loss of dendritic spines and reduced synaptic vesicles, is widespread in aging nervous systems (Hof & Morrison, 2004; Morrison & Baxter, 2012; Valdez et al., 2010). A global reduction in synaptic functions is thus thought to be a major cause for cognitive and behavioral decline related to aging (Morrison & Baxter, 2012). By contrast, the contribution to functional brain aging by sensory neurons, the first cells that detect and analyze environmental stimuli, remains incompletely defined.

To elucidate the role of sensory neurons in age‐related behavioral decline, we focus on the AFD neuron in the nematode *Caenorhabditis elegans*. AFD is the animal's major thermosensory neuron that senses temperature increase and drives thermotaxis behavior (Kimura, Miyawaki, Matsumoto, & Mori, 2004; Mori & Ohshima, 1995). On a thermal gradient, *C. elegans* animals migrate to the temperature at which they are previously cultivated with food (Hedgecock & Russell, 1975; Ito, Inada, & Mori, 2006). The response threshold and operating range of AFD are set by its temperature experience, and this plasticity of response property is recapitulated in cultured, dissociated AFD, suggesting that this is an AFD‐intrinsic property that does not depend on its synaptic connectivity (Kobayashi et al., 2016). Ablation of AFD disrupts temperature preference in migratory behaviors and results in animals that fail to track temperature (Mori & Ohshima, 1995). These studies highlight AFD as a critical site where acute temperature information is integrated with thermal experience to regulate thermotaxis behaviors.

The receptive ending is the subcellular compartment where a sensory neuron detects environmental cues and initiates sensory transduction. Similarly, temperature‐evoked neural activity in AFD is initiated from its receptive ending (Clark, Biron, Sengupta, & Samuel, 2006). The AFD receptive ending consists of a microtubule‐based cilium and actin‐rich microvilli (Doroquez, Berciu, Anderson, Sengupta, & Nicastro, 2014; Perkins, Hedgecock, Thomson, & Culotti, 1986). Molecules important for AFD activity, including the cyclic nucleotide‐gated cation channels composed of the TAX‐2 and TAX‐4 subunits, and receptor‐type guanylate cyclases GCY‐8, GCY‐18, and GCY‐23, are localized to the receptive ending (Coburn & Bargmann, 1996; Inada et al., 2006; Komatsu, Mori, Rhee, Akaike, & Ohshima, 1996). Mutations that impair structural integrity of the receptive ending result in defective sensory behaviors of *C. elegans* (Lewis & Hodgkin, 1977; Perkins et al., 1986), suggesting that the receptive ending is a crucial structure in the sensory neuronal circuits. While the genetic basis of receptive ending development in *C. elegans* has been studied in detail, it remains uncharacterized whether receptive endings undergo age‐dependent changes, and whether such changes, if existing, correlate with alterations in thermosensory behaviors.

In this study, we document progressive loss of microvilli and structural distortion of the AFD receptive ending during aging. In functional imaging, AFDs in aged animals show an expanded operating range, compared to prompt downregulation of calcium dynamics in young neurons. Furthermore, aged animals show cryophilia in thermotaxis assays but retain some experience‐dependent behavioral plasticity. We investigate the correlation between these aging hallmarks but find that in the *gcy‐8* guanylyl cyclase mutant, age‐related changes in AFD morphology and cryophilic behavior are reduced even with a prolonged AFD operating range. The lack of apparent correlation in structural and functional aging markers cautions against attempts to claim age‐dependent behavioral changes simply as results of malfunctions in primary sensory neurons.

## 2 RESULTS

### 2.1 Age‐dependent changes in the thermotaxis behavior of *C. elegans*

**FIGURE 1:**
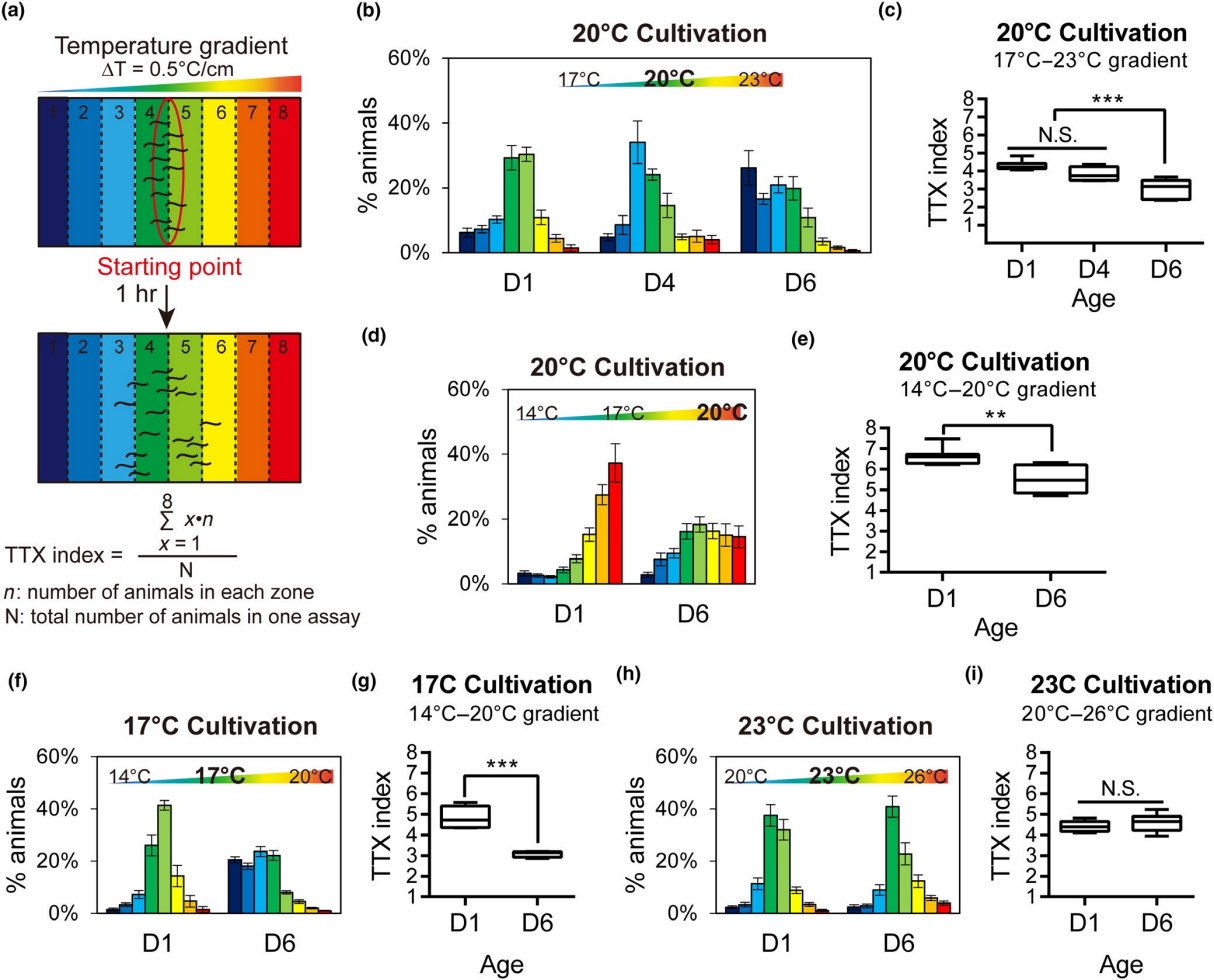
Age‐dependent changes in thermotaxis behavior. (a) Schematic diagram of the population thermotaxis (TTX) assay on a linear temperature gradient. (b, c) The distribution (b) and TTX index (c) of D1, D4, and D6 wild‐type animals on a temperature gradient of 17–23°C. *N* = 4–7 assays in each age cohort. (d, e) The distribution (d) and TTX index (e) of D1 and D6 wild‐type animals on a 14–20°C temperature gradient. *N* = 6–7 assays in each age cohort. (f, g) The distribution (f) and TTX index (g) of N2 animals cultivated at 17°C overnight before assay day. *N* = 6–7 assays in each age cohort. (h, i) The distribution (h) and TTX index (i) of wild‐type animals cultivated at 23°C overnight before assay day. *N* = 5–6 assays in each age cohort. Error bars are *SEM* (b, d, f, h), and box plots indicate mean, and the first and the third quartile (c, e, g, i). **p < .01, ***p < .001, N.S., not significant, one‐way (c, e) or two‐way ANOVA (g, i) followed by *Tukey* HSD multiple comparison test

To define the neural basis of age‐dependent decline in behavioral functions, we focus on *C. elegans* thermotaxis, a well‐established behavioral paradigm with its circuit basis partly resolved (Hedgecock & Russell, 1975; Ito et al., 2006; Mori & Ohshima, 1995). On a linear thermal gradient, young wild‐type animals migrate to a temperature zone corresponding to their prior cultivation temperature in the presence of food (Figure 1a,b). By laser ablation and genetic mutants, we have demonstrated that this associative thermotaxis behavior is critically dependent on AFD, a pair of bilaterally symmetric thermosensory neurons (Mori & Ohshima, 1995). Unlike young animals, we found that day 6 adult (D6) animals raised at 20°C migrated to temperature zones cooler (17°C) than their cultivation temperature (Figure 1b,c). When the thermal gradient in the test arena was centered at 17°C, young animals cultivated at 20°C successfully migrated toward 20°C, whereas D6 animals accumulated near 17°C (Figure 1d,e). D6 animals cultivated at 17°C migrated further down the temperature gradient (~14°C) (Figure 1f,g). Importantly, muscle structure and locomotion speed of D6 animals are not severely compromised (Glenn et al., 2004; Herndon et al., 2002; Hsu, Feng, Hsieh, & Xu, 2009; Liu et al., 2013). Furthermore, D6 animals exhibiting cryophilic behavior migrated over longer distances from the starting point than those with appropriate thermotaxis behavior (Figure 1b,c). Motor deficits are therefore unlikely to explain the altered thermotaxis of aged animals. These data suggest that aged animals encode or process the cultivation temperature, which results in cryophilia, migrating to temperature zones cooler than their conditioning cultivation temperature.

To investigate whether aged animals exhibit a cold shift in thermal preference across all temperature ranges, we cultivated animals at 23°C, before subjecting them to thermotaxis assays on linear temperature gradients. Interestingly, D6 animals cultivated at 23°C exhibited thermotaxis behavior indistinguishable from that of D1 animals (Figure 1h,i). Thus, age‐dependent changes in thermotaxis of D6 animals seem to occur specifically to animals raised at lower cultivation temperatures (17 and 20°C) but not at 23°C.

**FIGURE 2:**
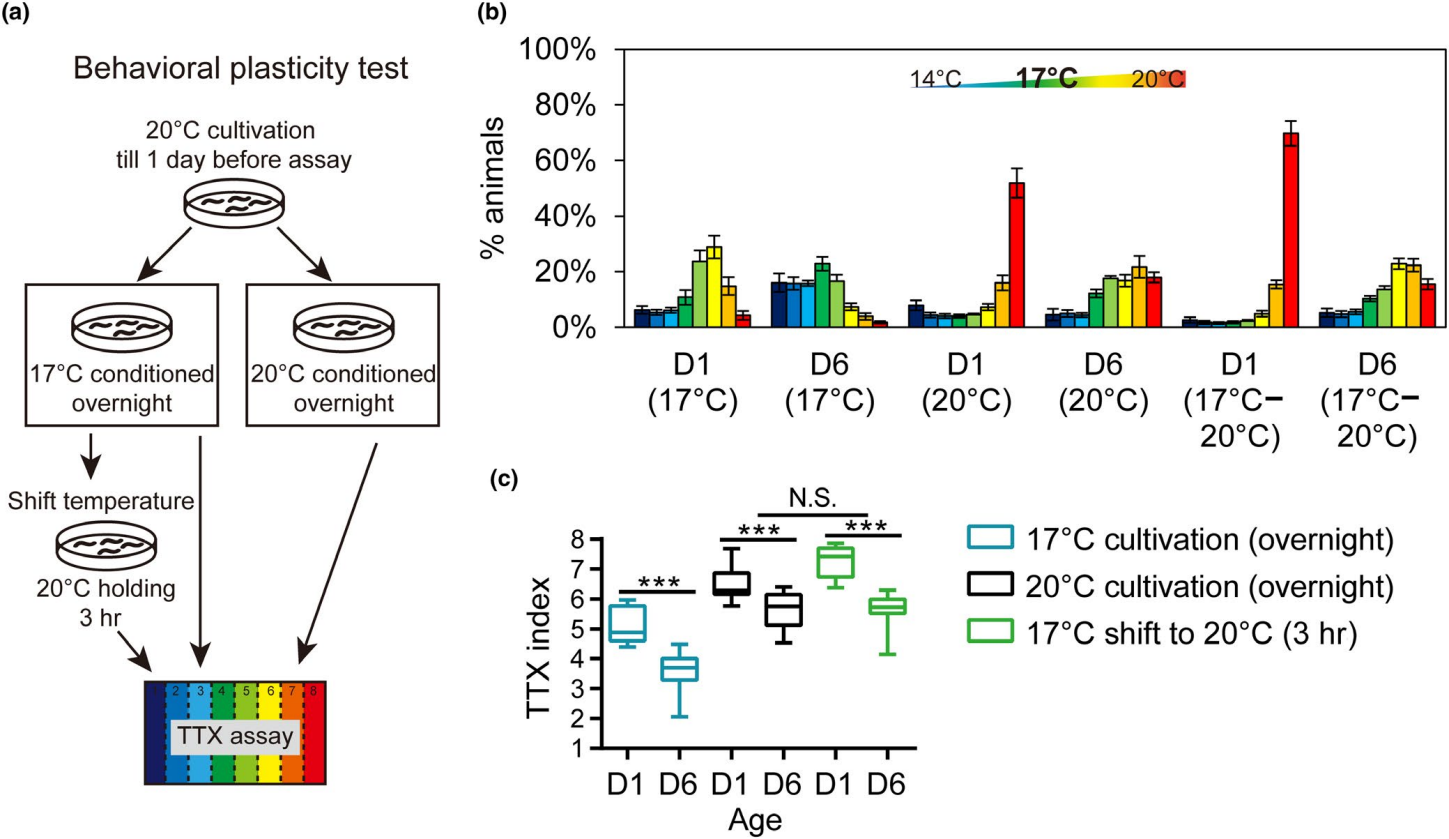
Behavioral plasticity test in young and aged *Caenorhabditis elegans*. (a) Schematic diagram of behavioral plasticity test using the population TTX assay. (b, c) The distribution (b) and TTX index (c) of wild‐type animals on the 14–20°C temperature gradient. *N* = 8–10 assays for each age–condition combination. Error bars are *SEM*, and box plots indicate mean, and the first and the third quartile. ***p < .001, N.S., not significant, two‐way ANOVA followed by *Tukey* HSD multiple comparison test

We also tested whether plasticity in thermotaxis behavior is affected in aged animals (Figure 2). Young *C. elegans* adults reset their temperature preference after shifting to a new cultivation temperature for as short as 3 hr, and this behavioral plasticity is associated with a corresponding shift in AFD operating range (Aoki et al., 2018). We found D6 animals remained competent in resetting thermotaxis behavior after an acute shift of cultivation temperature from 17 to 20°C, when corrected for their cryophilic behavior on thermal gradients under chronic 20°C cultivation (Figure 2b,c). Therefore, although aged animals display altered thermotaxis behaviors, they seem to retain appropriate plasticity in response to acute changes in thermosensory environments.

### 2.2 The operating range of AFD expands as the animals age

The threshold and operating range of AFD response is set by temperature experience, and this temperature‐dependent plasticity of AFD response property is at least partially independent of synaptic connection (Kobayashi et al., 2016). Therefore, AFD is potentially a significant contributor to thermotaxis changes during aging. Previous studies demonstrate that calcium dynamics in AFD are initiated from its sensory ending and that recordings of calcium transients from AFD soma reflect key features of temperature‐evoked responses recorded from sensory endings (Clark et al., 2006). These features include an onset of activation set by prior cultivation temperature and a phase‐locked response to temperature oscillations in a sinusoidal warming paradigm (Kimura et al., 2004).

**FIGURE 3:**
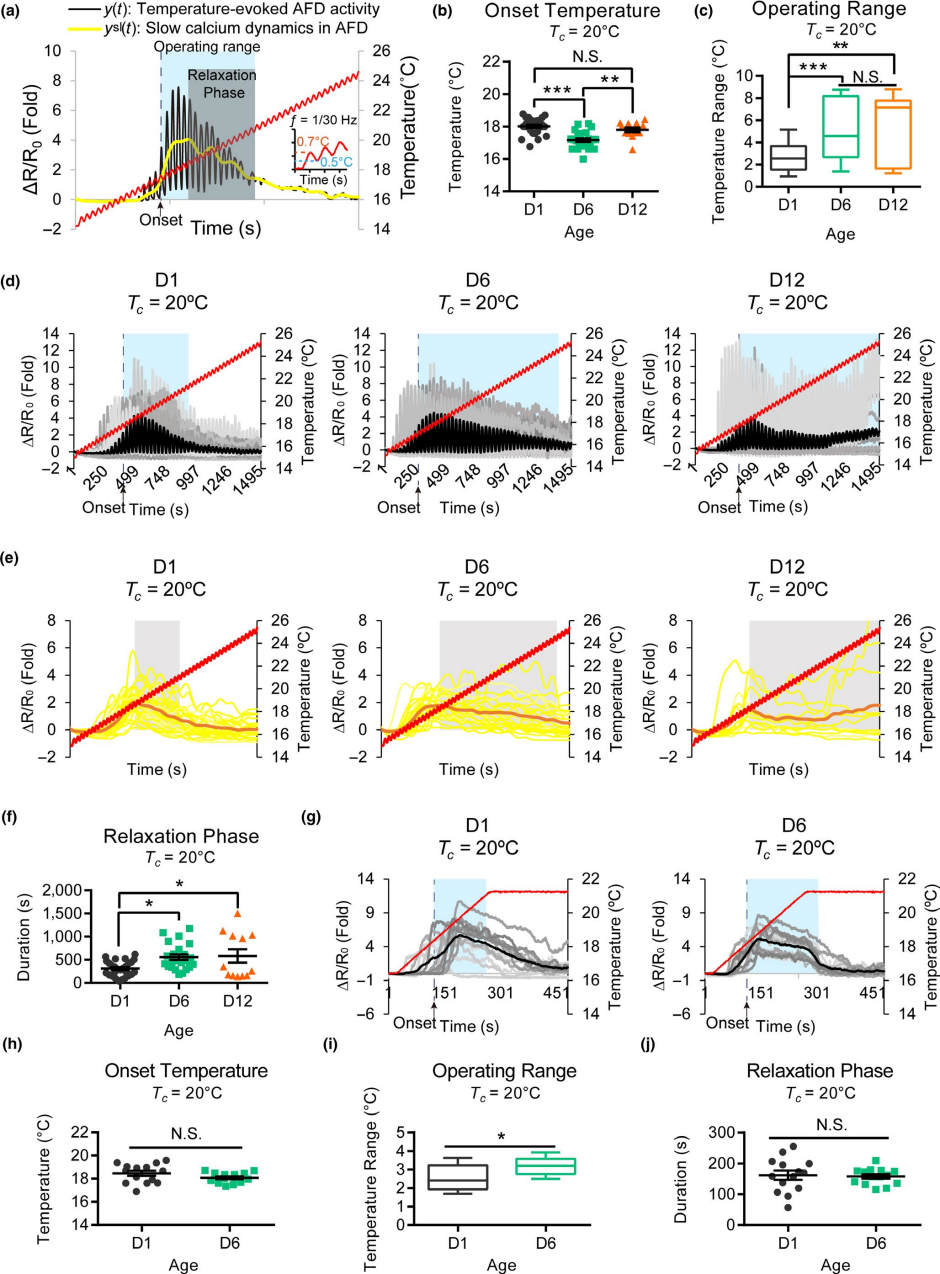
Age‐dependent changes in the temperature‐evoked calcium dynamics of AFD in animals cultivated at 20°C. (a) Quantitative analysis of temperature‐evoked calcium dynamics in AFD. The red curve represents a linear warming ramp from 15 to 25°C (0.4°C/min) with sinusoidal oscillation (1/30 Hz). The black trace represents the AFD calcium dynamics *y*(*t*). The yellow trace represents the slow calcium dynamics *y* ^sl^(*t*). The blue and gray shades indicate the operating range and relaxation phase of AFD responses, respectively. See also Figure S1 and Section 4 for details. (b) Quantification of temperature at AFD activation onset in *y*(*t*). Error bars = *SEM* **p < .01, ***p < .001, N.S., not significant, one‐way ANOVAfollowed by *Tukey* HSD multiple comparison test. (c) AFD operating range (the time interval from onset to the end of the relaxation phase) defined by *y* ^sl^(*t*). Boxes and bars are mean ± quartiles. **p < .01, ***p < .001, N.S., not significant, one‐way ANOVA with Dunnett's multiple comparison test. (d) Individual (gray) and averaged (black) calcium dynamics recorded from the AFD soma. (e) Slow calcium dynamics *y* ^sl^(*t*) for data shown in (d). Yellow: individual traces; orange: group average. Gray shades, the relaxation phase of *y* ^sl^(*t*). (f) Duration of the relaxation phase derived from data in (e). Error bars are *SEM*. N.S., not significant, *p < .05, one‐way ANOVA with Dunnett's multiple comparison test. For (b–f), *N* = 27, 23, and 12 neurons recorded in D1, D6, and D12 animals, respectively. (g) Individual (gray) and average (black) calcium dynamics recorded from the AFD soma under linear thermal stimulation from 16 to 21°C. Blue shades indicate the average operating range. *N* = 14 neurons for both D1 and D6 animals. (h–j) The onset temperature (h), operating range (i), and relaxation phase (j) calculated from data in (g). Error bars are *SEM*, and boxes are mean ± quartiles, respectively. *p < .05, *t* test

To characterize AFD response property during aging, animals at distinct age were subjected to oscillating thermal stimuli, and temperature‐evoked calcium dynamics in AFD were monitored using the genetically encoded calcium indicator, GCaMP5G (Figure 3a and Figure S1). This warming program allows visualization of individual AFD responses to temperature oscillation (Figure 3a). By recording calcium transients from the sensory ending and the soma of AFD in the same imaging frame, we confirmed that temperature‐evoked calcium transients in these two compartments were indeed nearly simultaneous in D1 animals (Figure S2). Simultaneous calcium responses between the sensory ending and soma were maintained in aged neurons, and most D6 and D12 AFDs showed robust phase‐locked calcium dynamics in response to thermal oscillation (Figure S2). As the GCaMP5G signal at sensory endings was weak, we analyzed sensory‐evoked responses by recording calcium dynamics in the AFD soma.

We found that the onset temperature for AFD calcium responses was significantly lower in D6 animals compared to that in D1 animals, although it did not differ significantly between D1 and D12 animals (Figure 3b). Calcium responses promptly decreased in D1 AFDs when the temperature rose above the point of maximal AFD response (Figure 3c‐f). By contrast, the decay in postmaximal responses (relaxation phase; see also Section 4) was blunted in D6 and D12 AFD neurons, resulting in wider operating ranges (Figure 3c‐f). Moreover, postmaximal responses of AFD in aged animals were persistent calcium transients phase‐locked to thermal oscillation instead of simply elevated, nonoscillating calcium levels. We speculate that AFDs in aged animals fail to transit temperature‐dependent activation from augmenting to decaying calcium responses that normally sharpens AFD activities around cultivation temperature in young animals. An analysis of cumulative calcium flux supports this idea, revealing a delay in reaching a plateau of total calcium flux in D6 and D12 animals (Figure S3). D12 AFD neurons showed a delay in the transition time from increasing to decreasing phases of calcium flux rates (Figure S3B–D).

Under our warming protocol (15–25°C), an expanded AFD operating range in aged animals cultivated at 20°C may suggest that these animals have prolonged duration in temperature‐evoked responses, or they are simply more sensitive to higher temperature (21–25°C). To test the first possibility, we stimulated AFDs with a linear warming program (16–21°C) that coincides with the optimal temperature range of AFD responses. Under this linear warming program, AFDs of D1 animals cultivated at 20°C activated at 18.46°C (the averaged onset temperature) and inactivated when the temperature is still increasing from 19 to 21°C (Figure 3g, D1 animals). By contrast, AFDs of D6 animals activated at 18.08°C (the averaged onset temperature) and sustained the activities with subtle decreases in calcium levels as long as the temperature kept rising from 19 to 21°C, but decreased promptly when the temperature stayed constant at 21°C (Figure 3g). This result suggests that the duration of temperature‐evoked AFD response in D6 animals itself is prolonged compared to those in D1 animals in response to the linear warming program. To evaluate the sensitivity of AFD to high temperatures in young and aged animals, we quantified the cumulative calcium flux of AFD responses at the high temperature range (21–25°C) by calculating the area between acute AFD activities (*y*(*t*)) and slow calcium dynamics (*y* ^sl^(*t*)) (Figure S3E and Section 4). We found that AFDs in D6 animals cultivated at 20°C kept responding drastically to high temperatures, resulting in a significantly larger accumulative area compared to those in D1 animals (Figure S3E). Therefore, our data indicate that the prolonged AFD response duration under optimal temperature range (Figure 3g‐i) and increased AFD responses at higher temperatures (Figure S3E) both contribute to the expansion of AFD operating range in D6 animals.

Age‐dependent cryophilic behavior is only observed in animals cultivated at 17 and 20°C but not at 23°C (Figure 1h,i). To understand the neural basis of this behavioral difference, we performed calcium imaging of AFD in D1 and D6 animals cultivated at 23°C. AFD calcium dynamics of D6 animals cultivated at 23°C showed similar onset temperature, operating range, and relaxation phase compared to those of D1 animals cultivated at the same temperature (Figure S4). Interestingly, the AFD operating range in either D1 or D6 animals cultivated at 23°C was significantly wider than that of D1 animals cultivated at 20°C (Figure 3c,d, and Figure S4A,C). Therefore, a wide AFD operating range alone is not sufficient to explain the cryophilic behavior in D6 animals cultivated at 20°C. Instead, past thermal experience and the evolution of AFD operating range are important factors that impact thermotaxis behaviors during aging.

### 2.3 Age‐dependent changes in AFD receptive endings

As AFD sensory endings show enrichment of the thermosensory guanylyl cyclases (Inada et al., 2006; Takeishi et al., 2016) and temperature‐evoked calcium responses are initiated in this compartment (Clark et al., 2006), we test whether AFD endings exhibit age‐dependent morphological changes, aiming to unravel the molecular basis for aging of AFD response properties and thermotaxis behavior.

**FIGURE 4:**
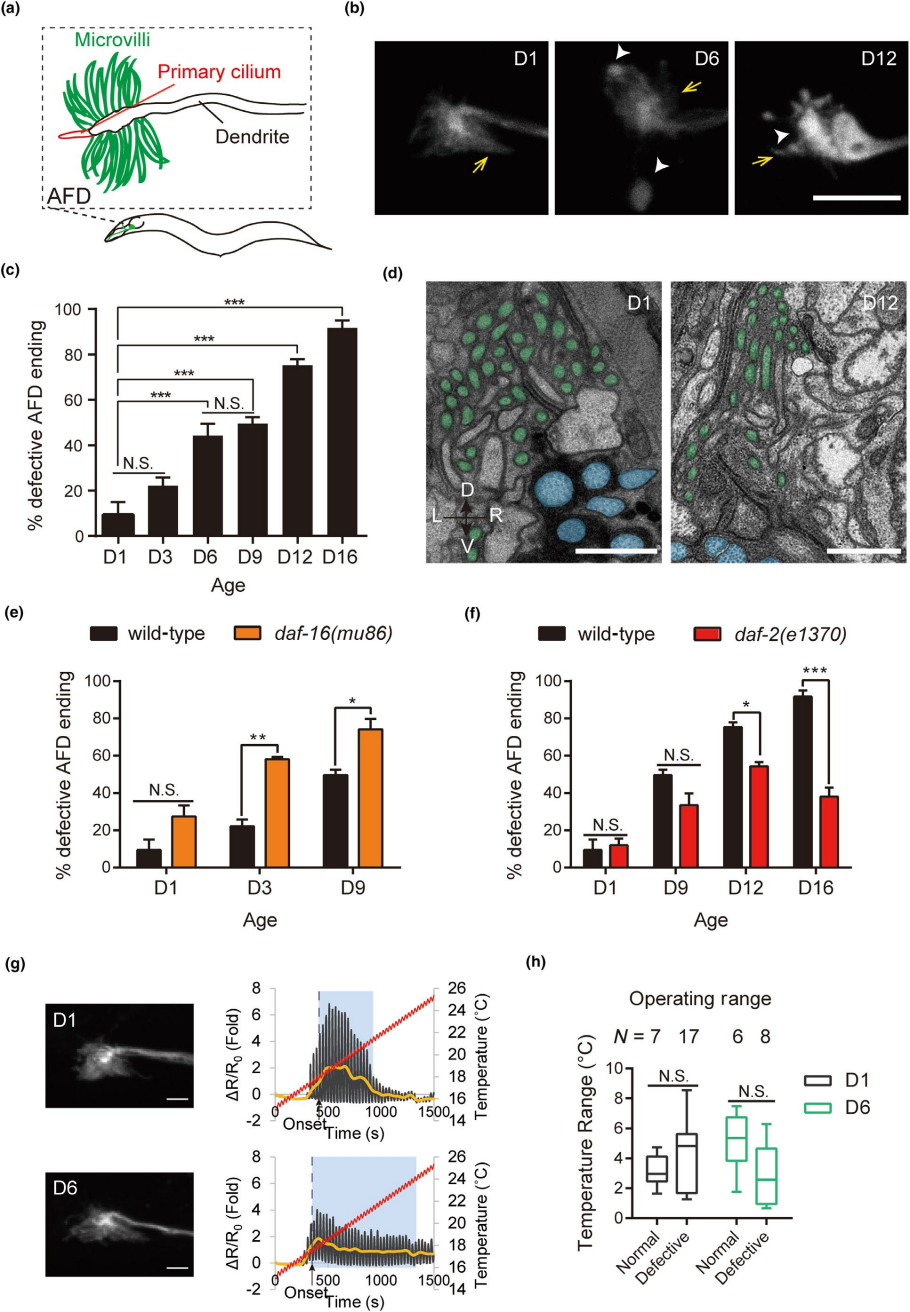
Age‐dependent changes in the AFD sensory ending. (a) Schematic diagram of the AFD sensory ending. (b) Representative confocal images of the AFD sensory ending during aging, marked by *oyIs18(Pgcy‐8::GFP)*. Arrows, microvilli; arrowheads, engorged structures in the central or domain of AFD ending. Scale bar = 5 µm. (c) Quantification of age‐dependent changes in the AFD ending. *N* = 3 independent experiments, with 12–31 AFD neurons per experiment. Total numbers of AFD neurons scored for each time points: D1 = 84, D3 = 67, D6 = 73, D9 = 59, D12 = 64, D16 = 59. These data are re‐plotted in (e) and (f), as well as in Figure 6c and Figure S5F. (d) Thin‐section electron micrographs of AFD ending in D1 and D12 animals. AFD microvilli are pseudocolored in green, and cilia of nearby amphid neurons are pseudocolored in blue. Scale bar = 1 µm. (e, f) Quantification of age‐dependent changes in AFD endings of the *daf‐16(mu86)* (e) and *daf‐2(e1370)* mutants (f). *N* = 3 independent experiments for each time point, with 13–28 neurons/experiment. Total numbers of AFD neurons scored: *daf‐16(mu86)*, D1 = 66, D3 = 66, D9 = 58; *daf‐2(e1370)*, D1 = 63, D9 = 61, D12 = 67, D16 = 60. For c, e, and f, error bars are *SEM* *p < .05, **p < .001, ***p < .001, N.S., not significant, two‐way ANOVA followed by *Tukey* HSD multiple comparison test. (g) Correlative analysis of AFD response property and sensory ending morphology. (Left) Projection confocal images of two representative AFD endings. (Right) Respective AFD response property. Scale bar = 2 μm. (h) The operating range of AFD sensory ending at D1 or D6. *N* = numbers of AFD neurons examined. *p < .05, N.S., not significant, Brown–Forsythe test

The AFD ending in young animals consists of a microtubule‐based cilium embedded in a nest of actin‐rich microvilli (Doroquez et al., 2014; Nguyen, Liou, Hall, & Leroux, 2014; Perkins et al., 1986) (Figure 4a). To test whether AFDs in aged animals undergo morphological changes, we used a number of cell‐specific fluorescent transgenes to examine AFD endings as well as dendrites (Figure 4b,c, and Table S2, Figure S5A,B). We found that AFD endings showed progressive deterioration with aging. Microvilli were reduced in D6 and D12 animals, with swelling of the distal ends of microvilli or engorgement of the central part of sensory ending (Figure 4b,c, and Table S2, Figure S5A,B). Consistent with these observations, serial thin‐section electron microscopy revealed microvilli loss and overall reduction in receptive ending size in D12 animal compared to those in D1 (Figure 4d; averaged microvilli number, D1, 43, *n* = 6 sides; D12, 23, *n* = 2 sides; average number of EM sections covering endings are 63 and 44 for D1 and D12, respectively).

To determine whether the changes we observed reflect a regulated aging process or are simply time‐dependent wear‐and‐tear, we examined animals with accelerated or delayed aging manifested as having shortened or prolonged lifespan, respectively (Kenyon, 2010). We found that age‐dependent changes of AFD endings were enhanced in the short‐lived *daf‐16*/FoxO mutant (Figure 4e) and were partially ameliorated in the long‐lived *daf‐2* mutant, which has a mutation in the gene encoding the insulin‐like growth factor 1 receptor (IGF1R) (Figure 4f). These findings show that age‐dependent changes in AFD endings are regulated by genes that control longevity, and suggest that they are likely morphological hallmarks of aging.

In addition to morphological changes in the sensory ending, we also found tortuosity, ectopic branching, and beading of the AFD dendrite in aged animals (Figure S5C,D), similar to those described for aged *C. elegans* mechanosensory neurons (Pan, Peng, Chen, & McIntire, 2011; Tank, Rodgers, & Kenyon, 2011; Toth et al., 2012). Sensory ending changes were also observed in animals with no gross dendrite deformation (Figure S5E,F), indicating that age‐dependent changes in the receptive ending and the dendrite can occur independently.

To determine whether changes in AFD response property correlate with structural changes of AFD ending, we recorded calcium transients and imaged the AFD ending subsequently in the same neuron, in D1 and D6 animals (Figure 4g). We found no correlation between the sensory ending morphology and the AFD operating range (Figure 4h). For example, many D6 AFD neurons with apparently intact sensory endings exhibited expanded operating range and prolonged relaxation phase (Figure 4g, h). By contrast, some D6 animals with deformed AFD endings displayed response properties indistinguishable from those of D1 control animals (Figure 4h). These observations suggest that altered response properties and changes of sensory endings can occur independently of each other in AFD neurons of aged animals. Unknown factors beyond gross changes in receptive ending morphology are likely to contribute to altered response properties in AFD of aged animals.

### 2.4 F‐actin disassembly and protein aggregation at aging sensory endings

Prolonged calcium responses in AFDs of aged animals may represent increased calcium influx upon thermal stimulation or decreased clearance of cytosolic calcium during relaxation phase. The three guanylyl cyclases, GCY‐8, GCY‐18, and GCY‐23, fuel cyclic GMP that gates the TAX‐2/TAX‐4 cation channel required for thermosensation (Komatsu et al., 1996,1999). GCY‐8, GCY‐18, and GCY‐23 are localized to the actin‐rich microvilli, whereas TAX‐2 and TAX‐4 subunits are enriched in the cilium (Coburn & Bargmann, 1996; Inada et al., 2006; Komatsu et al., 1996; Nguyen et al., 2014). While missing one of the three guanylyl cyclases has minimal effects on AFD responses to thermal stimuli, elimination of all three abolishes AFD responses (Kuhara, Ohnishi, Shimowada, & Mori, 2011; Ramot, MacInnis, & Goodman, 2008; Takeishi et al., 2016). Ectopic expression of individual thermosensory guanylyl cyclase confers thermal sensitivity to characteristic temperature ranges (Takeishi et al., 2016). The exact stoichiometry of these three molecules and their contribution to AFD response property during aging remain unexplored. Therefore, we investigate how actin structures and thermosensory GCY proteins change in AFDs of aged animals.

**FIGURE 5:**
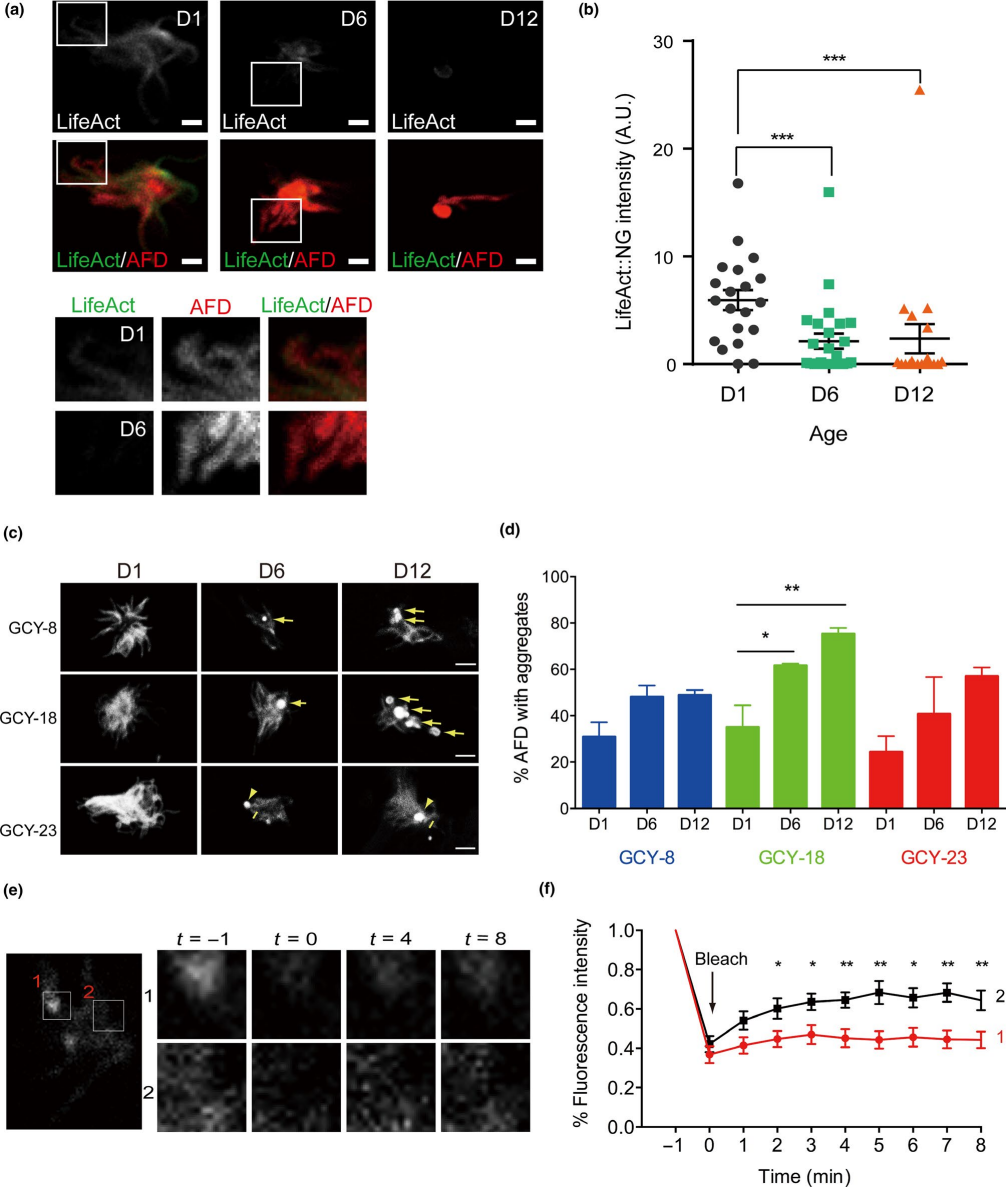
Loss of F‐actin and aggregation of thermosensory guanylyl cyclases during aging. (a) Confocal images of the AFD ending. F‐actin and the AFD neuron are labeled in green and red, respectively, by the transgene *twnEx485(Pgcy‐8::LifeAct::NG, Pgcy‐8::mCherry*). Boxed regions are highlighted in the bottom panels. Scale bar = 3 µm. (b) Quantification of LifeAct::NG signals. Bars are mean ± *SEM* D1, *N* = 20, D6, *N* = 25, D12, *N* = 19. ***p < .001, Mann–Whitney *U* test. (c, d) Confocal images of GCY‐8, GCY‐18, and GCY‐23 localization during aging (c) and the quantification of protein aggregates (d). Three independent experiments for each genotype at time point, with 14–20 AFD neurons in one experiment. Total number of AFD neurons scored: GCY‐8, D1 = 42, D6 = 54, D12 = 45; GCY‐18, D1 = 49, D6 = 47, D12 = 53; GCY‐23, D1 = 44, D6 = 53, D12 = 58. *p < .05, **p < .01, one‐way ANOVA with Bonferroni's multiple comparison test. (e, f) FRAP for GCY‐8 aggregates. 1 and 2 in (e) indicate aggregated and diffuse GCY‐8::GFP signals in the same AFD sensory ending, respectively. (f) Quantification of FRAP. *N* = 14 pairs of FRAP experiments, *p < .05, **p < .01, Mann–Whitney *U* test

We first examined F‐actin assembly in AFD endings using NeonGreen (NG)‐tagged LifeAct, a short peptide that binds F‐actin (Riedl et al., 2008). In D1 animals, LifeAct::NG signal specifically labeled individual microvilli in AFD endings (Figure 5a,b). In D6 animals, LifeAct::NG signal was markedly reduced in microvilli, and it often accumulated at the center of AFD ending. F‐actin was nearly completely absent from the microvilli of AFD endings in D12 animals (Figure 5a,b).

We next examined whether age‐related changes occur to the three thermosensory guanylyl cyclases at AFD endings. Consistent with our prior study (Inada et al., 2006), GFP‐tagged GCY‐8, GCY‐18, and GCY‐23 showed exclusive localization to the AFD ending, and this pattern was maintained in AFDs of aged animals (Figure 5c,d). In agreement with prior reports showing increased protein aggregation in aged *C. elegans* (David et al., 2010), we found progressive aggregation of GCY‐8, GCY‐18, and GCY‐23 proteins in D6 and D12 animals, while control animals expressing GFP only did not show such aggregation (Figure 4b). We confirmed the nature of protein aggregation by fluorescence recovery after photobleaching (FRAP), suggesting limited protein mobility of aggregated GCY‐8::GFP (Figure 5e,f). We speculate that loss of F‐actin, aggregation of thermosensory guanylyl cyclases, and other factors contribute to altered sensory‐evoked calcium dynamics in AFDs of aged animals.

### 2.5 Loss of the GCY‐8 guanylyl cyclase decreases morphological and behavioral aging

**FIGURE 6:**
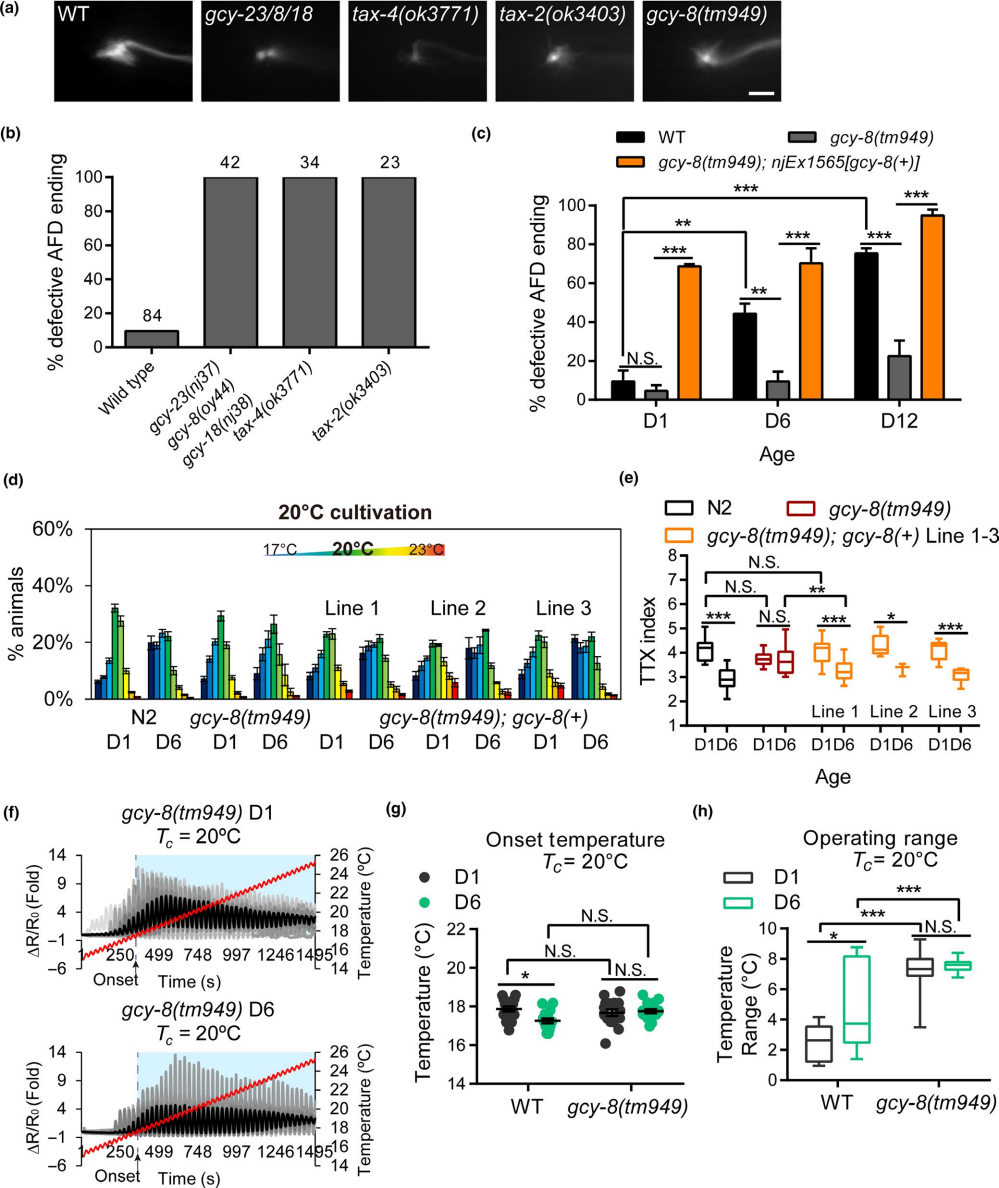
Loss of GCY‐8 modifies AFD sensory ending morphology and thermotaxis behavior in aging. (a) Representative fluorescent images of AFD ending in D1 animals of respective genotypes. Scale bar = 2 µm. (b) Quantification of age‐dependent changes in AFD ending of D1 animals. N, the numbers of AFD neurons scored. (c) Quantification of age‐dependent changes in AFD endings of the *gcy‐8(tm949)* mutant. *N* = 3–4 independent experiments for each age group, with 9–29 AFD neurons per experiments. Total numbers of animals scored at each time point: *gcy‐8(tm949)*, D1 = 74, D6 = 68, D12 = 49. *gcy‐8(tm949); njEx1565[gcy‐8(+)]*, D1 = 92, D6 = 78, D12 = 40. Wild‐type data are re‐plotted from those in Figure 4c. Error bars are *SEM* **p < .01, ***p < .001, N.S., not significant, two‐way ANOVA followed by Tukey HSD multiple comparison test. (d, e) The distribution (d) and TTX index (e) of D1 and D6 wild‐type, the *gcy‐8(tm949)* mutant, and three independent *gcy‐8* rescue transgenic lines on a 17–23°C temperature gradient. *N* = 7–29 assays for N2 and *gcy‐8(tm949)* animals and *N* = 3–18 assays for GCY‐8 rescue lines. (f) Individual (gray) and average (black) calcium dynamics recorded from the AFD soma of *gcy‐8(tm949)* animals under oscillating thermal stimulation ramping from 15 to 25°C. Blue shades indicate operating ranges. (g, h) The onset temperature (g) and operating range (h) of D1 and D6 wild‐type or *gcy‐8(tm949)* animals in (f). Error bars are *SEM* Boxes represent mean the first and the third quartile. *p < .05, ***p < .001, N.S., not significant, two‐way ANOVA followed by Tukey HSD multiple comparison test

We previously showed that neural activity maintains the structural integrity of *C. elegans* neurons during aging (Jiang et al., 2015; Pan et al., 2011). Activation of AFD requires cyclic nucleotide‐gated (CNG) cation channel subunits TAX‐2 and TAX‐4, as well as three receptor‐type guanylyl cyclases: GCY‐8, GCY‐18, and GCY‐23 (Kimura et al., 2004; Takeishi et al., 2016). The *tax‐2*, *tax‐4*, or the *gcy‐18 gcy‐8 gcy‐23* triple mutants showed complete penetrance of defective AFD endings at D1, suggesting that intact AFD activity is essential for AFD ending development (Figure 6a,b). As AFD responses were present in most aged animals given a high incidence of sensory ending changes (Figures 3d and 4c), we think it is unlikely that the absence of AFD responses in the *tax‐2*, *tax‐4*, or the *gcy‐18 gcy‐8 gcy‐23* triple mutants is simply a consequence of disrupted sensory endings in these animals.

To understand the role of thermosensory guanylyl cyclases in age‐dependent modulation of AFD ending morphology and thermotaxis behavior, we examined strains with mutations in the *gcy‐8* or *gcy‐18* genes. The *gcy‐8(tm949)* mutant with null cyclase activity had intact AFD development. Surprisingly, age‐related changes in the AFD endings of D6 and D12 *gcy‐8(tm949)* animals were far less frequent compared to those of age‐matched controls (Figure 6c). These effects of *gcy‐8(tm949)* on AFD ending morphology were mirrored in the thermotaxis behavior: D6 *gcy‐8(tm949)* animals showed intact thermotaxis and were indistinguishable from D1 wild‐type animals regarding this behavior (Figure 6d,e). Expression of *gcy‐8* genomic DNA in AFD re‐instated the cryophilic behavior in the aged *gcy‐8(tm949)* mutant (Figure 6d,e). These data suggest that *gcy‐8*, rather than unknown background mutations, is responsible for the age‐dependent changes in the thermotaxis behavior. By contrast, the *gcy‐18(nj38)* loss‐of‐function mutation did not have discernable effects on age‐related cryophilic behavior (Figure S6A,B), suggesting that different guanylyl cyclases play distinct roles in modulating thermotaxis behavior during aging. It had been shown that excessive GCY‐8 activity, conferred by a gain‐of‐function *gcy‐8* mutation, results in shortened AFD microvilli and cryophilic behavior (Singhvi et al., 2016). The phenotypic similarity between the *gcy‐8* gain‐of‐function mutant and D6 wild‐type, together with the effects on aging phenotypes conferred by loss of *gcy‐8*, raises the intriguing possibility that dysregulated GCY‐8 guanylyl cyclase activities contribute to age‐dependent changes in AFD ending and thermotaxis behavior. Supporting this speculation, re‐expressing a PCR‐amplified *gcy‐8* genomic fragment containing its promoter, coding sequence as well as the 3’UTR in the *gcy‐8(tm949)* mutant, increased the penetrance of morphological changes in AFD sensory ending of D1, D6, and D12 adults compared to *gcy‐8(tm949)* mutant (Figure 6c). The rescued *gcy‐8(tm949)* mutant animals were behaviorally wild‐type (Figure 6d,e), suggesting that changes in thermotaxis behavior during aging in the putative *gcy‐8* strain are caused by the *gcy‐8(tm949)* mutation, instead of unknown background mutations in this strain. However, as significantly increased AFD morphological defects in the rescued *gcy‐8(tm949)* mutant animals were also evident in D1, we could not exclude the possibility that some of these morphological changes are artifacts due to transgene overexpression.

To gain further insight into the neural mechanisms of the absent cryophilic behavior in aged *gcy‐8(tm949)* mutant, we monitored temperature‐evoked calcium dynamics in AFD of D1 and D6 *gcy‐8* animals. Unexpectedly, our experiments showed that the operating range of AFD response was significantly expanded in D1 *gcy‐8(tm949)* animals, mainly due to a prolonged relaxation phase, but also in a small part to slightly lower onset temperature (Figure 6f–h). However, unlike the wild‐type, the relaxation phase of AFD responses was not further prolonged in D6 *gcy‐8* mutant animals (Figure 6h). These results were reminiscent of the observations made in wild‐type animals cultivated at 23°C, where expanded AFD operating range was documented in both D1 and D6 animals without cryophilic thermotaxis behavior (Figure 1h,i and Figure S4). These results argue against the simple model that prolonged AFD relaxation phase per se is causally related to cryophilic behavior. Rather, we speculate that thermal experience and age‐dependent evolution of operating range may engage other molecular pathways in AFD to control thermotaxis behaviors during aging.

## 3 DISCUSSION

### 3.1 The lack of apparent correlation between prolonged AFD operating range and altered thermotaxis behaviors in aged animals

Several lines of evidence implicate AFD as a major site of encoding and processing of temperature information that instructs thermotaxis behavior via patterned calcium transients. First, aberrant calcium dynamics or calcium‐dependent signaling in AFD has been shown to result in abnormal thermotaxis behaviors (Kuhara, Inada, Katsura, & Mori, 2002). Second, AFD calcium responses recorded from freely moving animals are sufficient to reconstruct the thermal environment and trajectories of an animal navigating on a temperature gradient (Tsukada et al., 2016). Third, recent work from one of our laboratories and others suggests that AFD generates distinct synaptic signals that are valued in the AIY interneuron to drive appropriate thermosensory behavior (Hawk et al., 2018; Kuhara et al., 2011; Nakano et al., 2020). While the calcium imaging results have hinted at the role of AFD in directing thermotaxis behaviors of young adult *C. elegans*, how aging affects AFD activities and influences thermotaxis behaviors is still unclear. Here, we find that AFDs of D6 animals show significantly earlier activation onset upon warming as well as hypersensitivity to high temperatures, resulting in prolonged calcium transients over wider temperature ranges. We speculate that altered calcium dynamics in AFDs of aged animals may result in faulty representation of temperature information, which affects the animal's thermal memory and contributes to the cryophilic behaviors associated with aging. However, we find no evidence of correlation between prolonged temperature‐evoked AFD activities and the cryophilic thermotaxis behaviors in aged animals. Age‐dependent cryophilic behavior is absent in wild‐type animals cultivated at 23°C and in the *gcy‐8* guanylyl cyclase mutant cultivated at 20°C. In both cases, prolonged AFD operating range is already present in young adults. Therefore, a prolonged AFD operating range alone is not sufficient to account for cryophilic behavior during aging. Rather, the evolution of AFD response properties during aging may be an important factor that influences thermotaxis behavior of aged animals. A wild speculation is that when the AFD operating range is similar between young and aged animals, no age‐dependent cryophilic behavior is observed. AFD responses are part of the encoding system that reconstructs the thermal environment in the primary sensory neuron. Altered response property in aged animals may result in encoding of thermal environment that differs from that made by AFDs in young animals, which impacts thermotaxis behavior with other factors.

The physiological significance of age‐related cryophilic behavior remains elusive. Exotherms including nematodes regulate their body temperatures by moving toward thermal environments that are optimal for their physiology and metabolism. A cold‐shifted behavior in aged animals may be an adaptation for the animals to counteract the adverse effects of aging, as low temperature is associated with delayed aging and extended lifespan (Xiao et al., 2013). Alternatively, cryophilia may be behavioral aberrancy that results from deficits in the processing of thermal information in aged animals.

### 3.2 The GCY‐8 guanylyl cyclase and age‐dependent changes in AFD ending morphology and thermotaxis behavior

In an attempt to dissect the molecular basis of age‐dependent changes in the sensory ending shape and response property of AFD, we identify disassembly of F‐actin and aggregation of thermosensory guanylyl cyclases in AFD endings of aged animals. Although loss of the GCY‐8 guanylyl cyclase activity decreases morphological changes of the AFD ending and cryophilic behaviors in aged animals, the role of *gcy‐8* remains unsettled, as morphological rescue experiments are complicated by technical issues such as gene overexpression from multi‐copy transgenic arrays. Single‐copy transgenes that express *gcy‐8* at near‐physiological level may help to solve this issue. As GCY‐8 is localized to the microvilli (Nguyen et al., 2014), disorganized or shrunken microvilli associated with F‐actin loss may lead to redistribution of GCY‐8 and other molecules important for thermosensory transduction, such as the G protein‐coupled receptor SRTX‐1 and the CNG channel subunits TAX‐2 and TAX‐4 (Colosimo et al., 2004; Nguyen et al., 2014), which potentially change the spatiotemporal patterns of calcium dynamics. An intriguing hypothesis is that patterned AFD calcium dynamics evoked by thermal stimuli specify the valence and magnitude of synaptic signals to AIY that instruct thermotaxis behaviors (Kuhara et al., 2011; Hawk et al., 2018; Nakano et al., 2020). However, it should be noted that we did not find a correlation between deformed microvilli organization and prolonged calcium responses in AFDs of aged animals, although this conclusion should be cautioned and confirmed in future studies due to the small sample size of our experiments. It is also unknown whether diminished F‐actin assembly and GCY‐8 aggregation are causally related, although it is previously shown that expression of WSP‐1/Wiskott–Aldrich syndrome protein (WASP), a well‐studied F‐actin organizer, partially mitigates microvilli defects caused by excessive GCY‐8 activity (Singhvi et al., 2016).

In summary, our data highlight primary sensory neurons as a substrate of aging and identify hallmarks in sensory ending morphology and neural activity associated with senescence. Important future challenges include the identity of molecules that regulate the relaxation phase of evoked AFD responses, the mechanistic link between thermosensory calcium dynamics and synaptic signals from AFD, and the signaling pathways through which aging influences the structure and response property of primary sensory neurons. Elucidation of these enigmas will pave the way for the development of novel strategies that counteract neuronal aging and restore behavioral competence in senescence.

## 4 EXPERIMENTAL PROCEDURES

### 4.1 *C. elegans* strains and genetics

*Caenorhabditis elegans* strains were cultivated as previously described (Brenner, 1974). N2 (Bristol strain) was used in all experiments as a wild‐type or control. All animals were collected at the fourth larval stage (L4) and scored at indicated days in adulthood. All strains are maintained at 20°C in most experiments as described unless indicated otherwise. A list of strains and transgenes used in this study is available in Table S1.

### 4.2 Molecular biology and germline transformation

A 810‐bp fragment of the *gcy‐8* promoter that is insensitive to temperature is used for AFD‐specific expression in the current study, which ensures that transgene expression level is not discernibly altered by temperature change (Chen et al., 2016). Sequences that encode LifeAct (72 bp), mNeonGreen (915 bp), GCaMP5G (1,353 bp), and mCherry (711 bp) were engineered to be expressed from this *gcy‐8* promoter for F‐actin visualization or calcium imaging in AFD. Constructs used for generating transgenes of the *twnEx* series use the pPD95.77 fire vector as their backbone and listed in the key resource table. Germline transformation by microinjection was performed as previously described (Mello, Kramer, Stinchcomb, & Ambros, 1991).

### 4.3 Electron microscopy

L4 + 24 hr (Day 1) or Day 12 animals were prepared for electron microscopy using standard methods (Lundquist, Reddien, Hartwieg, Horvitz, & Bargmann, 2001). The transverse ultrathin sections of *C. elegans* (70 nm) were serially collected from the tip of the nose using a Leica Ultracut UCT Ultramicrotome. Electron microscopy images were acquired using a FEI Tecnai G2 Spirit BioTwin transmission electron microscope operating at 120 kV with a 4K digital camera.

### 4.4 Fluorescence microscopy and quantification of fluorescence intensity

AFD endings of adult *C. elegans* at indicated age are examined by Confocal Imaging System LSM 700 and LSM 880 (Carl Zeiss) under the 63× and 100× objectives (NA = 1.4). z‐stack projection images are used for assessment of sensory ending morphology or F‐actin abundance. Changes of AFD ending are defined as a partial or complete absence of microvilli, swollen ciliary structure, or abnormal branching at the sensory terminal, as detailed below. In D1 wild‐type animals, the peripheral territory of the AFD ending is densely decorated by microvilli without gaps. Partial absence of microvilli is suggested by gaps of microvilli occupancy, and complete absence of microvilli is suggested by failure to reveal microvilli by AFD‐specific fluorescent transgenes. Swollen ciliary structure indicates engorgement of the central domain of the AFD ending, where the cilium is located. As the AFD dendrite does not have any collateral branches, any branching seen at or near the sensory ending revealed by fluorescent transgenes is abnormal. We excluded the possibility of dendrite branching being artifacts caused by the fluorescent transgenes, as none of the GFP or mCherry transgenes that we used caused AFD dendrite branching in young wild‐type animals. Using ImageJ, the F‐actin signal is quantified as pixel density of NeonGreen fluorescence of the AFD sensory ending defined by *Pgcy‐8::mCherry* expression. Therefore, the LifeAct::NeonGreen pixel density in A.U. estimates F‐actin abundance per unit area specifically in the AFD sensory terminal.

### 4.5 Calcium imaging

Temperature control for calcium imaging of AFD was described previously (Kobayashi et al., 2016). We apply a linear warming stimulus from 15 to 25°C with superimposed sinusoidal oscillation of a 0.7°C warming step for 15 s followed by a 0.5°C cooling step for 15 s. Each single recording session is 25 min. D1 neurons were recorded the same day as control for all recordings of D6 and D12 neurons. Epifluorescent images were acquired under the 40X objective (NA = 0.9) of the upright BX61WI microscope (Olympus) at 1 frame/second with 400 ms of exposure time. Analysis of fluorescent intensity was performed in MetaMorph (Molecular Devices).

### 4.6 Analysis of calcium dynamics

mCherry is co‐expressed with GCaMP5G signal under the same promoter as a reference to correct for signal changes from minor focal plane shift during calcium imaging. As mCherry signal from the AFD sensory ending is weak and labile to photobleaching, the analysis of calcium transients recorded from the sensory ending uses only data of GCaMP5G signals. GCaMP5G/mCherry ratio in AFD soma defines *R*(*t*), a time function of AFD response. AFD calcium dynamics, *y*(*t*), is further calculated as:

$$y\left(t\right)=\frac{R\left(t\right)}{R_{0}}$$

where $\Delta R\left(t\right)=R\left(t\right)-R_{0}$. The baseline, *R*_0_, is defined as the averaged value of the first 10 frames in *R*(*t*) where the temperature is constant before warming. *y*(*t*) consists of two dynamics: the fast dynamics phase‐locked to temperature oscillation, and *y* ^sl^(*t*), the slow dynamics describing long‐term trend of calcium transients. *y* ^sl^(*t*) is extracted by a low‐pass Butterworth filter that attenuates the signals with frequencies higher than the sinusoidal temperature oscillation (1/30 Hz) from *y*(*t*). Onset of activation is defined as the time required for *y*(*t*) to reach the value of $\frac{y_{\max}}{e}$ from *t* = 0, where *y*_max_ is the maximal value of *y*(*t*), and *e* is Napier's constant. Relaxation phase is the time required for *y* ^sl^(*t*) to decrease till the value of $\frac{y_{\max}^{\text{sl}}}{e}$ after reaching its maximal value, $y_{\max}^{\text{sl}}$. The operating range of AFD is defined as the temperature range between the onset and the end of the relaxation phase.

### 4.7 Curve fitting of accumulative AFD calcium flux

The accumulative calcium flux, *S*(*t*), is the accumulative area bounded by the curves of *y*(*t*) and *y* ^sl^(*t*). We utilize the third‐order spline interpolation to interpolate three additional time points evenly between each interval of two frames (1 s) while keeping the shape similar to the raw data. We then calculate the accumulative area along the recording time series by Simpson's rule in numerical integration:

(1) $$S\left(t\right)=\int_{0}^{t}\left|y\left(t^{\prime }\right)-y^{\text{sl}}\left(t^{\prime }\right)\right|dt^{\prime }.$$

*S*(*t*) reflects the amount of calcium flux from slow calcium dynamics. We assume that the time derivative of cumulative calcium flux in AFD, $\frac{dS\left(t\right)}{\mathrm{dt}}$, is regulated by a linear increasing factor and an exponential decreasing factor. The increasing factor is proportional to current value of *S*(*t*), and the decreasing factors is exponentially decreased with time:

(2) $$\frac{dS\left(t\right)}{\mathrm{dt}}=ASe^{-\gamma t}$$

where *A* and *γ* are control parameters. The solution of the differential equation is written by Gompertz function, an exponentiated exponential function (Easton, 1978; Narinç, Öksüz Narinç, & Aygün, 2017).

(3) $$S\left(t\right)=\alpha \beta^{e^{-\gamma t}}$$

where the *α*, *β*, and *γ* are constants in Gompertz function. The inflection point (*t_s_*) of *S*(*t*) indicates the transition time from the activation phase to the relaxation phase in AFD accumulative calcium flux:

(4) $$t_{s}=\frac{1}{\gamma }\log\left(-\log\beta \right).$$

### 4.8 Thermotaxis assay

Population thermotaxis assays are performed as previously described (Ito et al., 2006). Each assay plate (140 × 100 × 14.5 mm, No. 2 dish, Eiken Chemical Co.) contains 18 ml of the assay medium (2% agar, 0.3% NaCl, and 25 mM pH 6.0 potassium phosphate), making a thin layer of agar for efficient temperature transmission. The assay plates are air‐dried at room temperature for 30 min before use. A stable, linear thermal gradient is established across a 600 mm‐long aluminum platform, with its two ends in water baths at 7 and 35°C, respectively. A thin aluminum (135 × 95 × 1 mm) plate is placed between the assay plate and the aluminum platform with water as a mediator to optimize temperature transmission. Synchronized adult animals precultivated at 20°C are transferred onto the center of the thermotaxis plate by washing with NG buffer (0. 3% NaCl, 1 mM CaCl_2_, 1 mM MgSO_4_, and 25 mM pH 6.0 potassium phosphate). The distribution of animals is recorded 1 hr later after stopping the animals with chloroform. We assign a score of 1 (coolest) to 8 (warmest) on the basis of temperature zones on the thermotaxis plates. The thermotaxis (TTX) index was calculated as:

(5) $$\text{TTX index}=\frac{\sum_{x=1}^{8}x\times n}{N}$$

where *n* is number of animals in each zone and *N* is the total number of animals on the assay plate. 100–300 animals are counted in each assay.

### 4.9 Statistical analysis

All the statistical analyses are performed in Excel (Microsoft) and Prism (GraphPad). Sample numbers are specified in respective Figure Legends. Mann–Whitney *U* test is used to determine the statistical significance of F‐actin signals. ANOVA and Brown–Forsythe tests with appropriate corrections are used to determine the statistical significance of data in sensory ending morphology, neural activity, and thermotaxis assays.

## CONFLICT OF INTEREST

The authors declare no competing financial interest.

## AUTHOR CONTRIBUTIONS

T.‐T.H., H.J.M., Y.T., I.M., and C.‐L.P. designed research; T.‐T.H., H.J.M., Y.T., A.S., R.‐T.S., Y.L., and C.‐L.P. performed research; T.‐T.H., H.J.M., Y.T., A.S., R.‐T.S., Y.L., S.S., I.M., and C.‐L.P. analyzed data; T.‐T.H., H.J.M., Y.T., A.S., S.S., I.M., and C.‐L.P. wrote the paper.

## Supporting information

Supplementary Material

## Data Availability

This article is exempt from Mandates Data Policy as it was originally submitted before the adoption of the policy.
