# Corrigendum: Age‐dependent changes in response property and morphology of a thermosensory neuron and thermotaxis behavior in *Caenorhabditis elegans*

**DOI:** 10.1111/acel.13271

**Published:** 2020-11-22

Tzu‐Ting Huang, Hironori J. Matsuyama, Yuki Tsukada, Aakanksha Singhvi, Ru‐Ting Syu, Yun Lu, Shai Shaham, Ikue Mori, Chun‐Liang Pan. *Aging Cell*, **19**, e13146. https://doi.org/10.1111/acel.13146

In the original publication of the article by Huang, T.T. et al. (2020), the authors would like to correct the following:

**1. In section 4.6, the definition of onset:**

**Original:**

Onset of activation is defined as the time required for *y*(*t*) to reach the value of $\frac{y_{\max}}{e}$ from *t* = 0, where $y_{\max}$ is the maximal value of *y*(*t*), and e is Napier's constant.

**Correction:**

Onset of activation is defined as the time required for *y*(*t*) to reach the value of $\frac{y_{\max}}{2}$ from *t* = 0, where $y_{\max}$ is the maximal value of *y*(*t*).

**2. In the legend of Figure 3c:**

**Original:**

(C) AFD operating range (the time interval from onset to the end of the relaxation phase) defined by y^sl^(t).

**Correction:**

(C) AFD operating range (the temperature range between the onset and the end of the relaxation phase) defined by *y* ^sl^(*t*).

**3. Figure 4h, N numbers of D1 group in the Operating Range:**

**Original:**

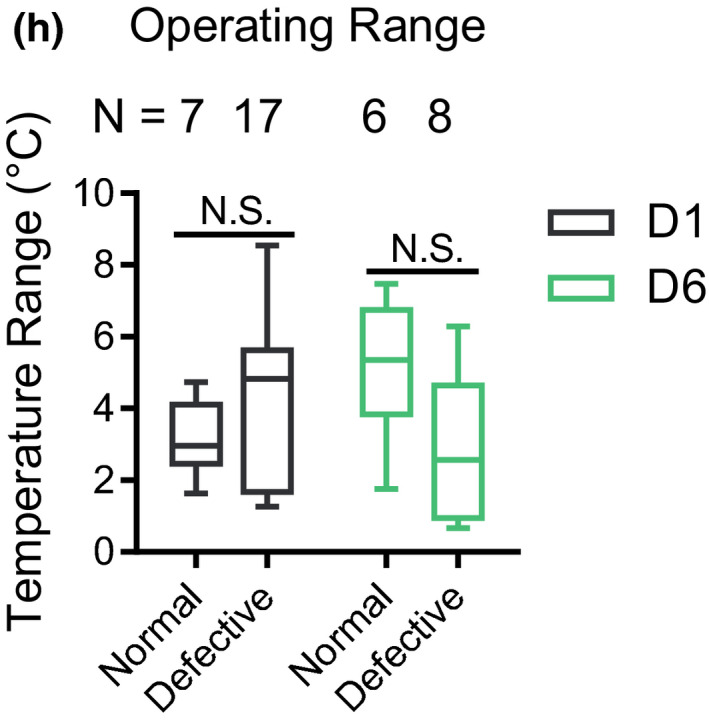

**Correction:**

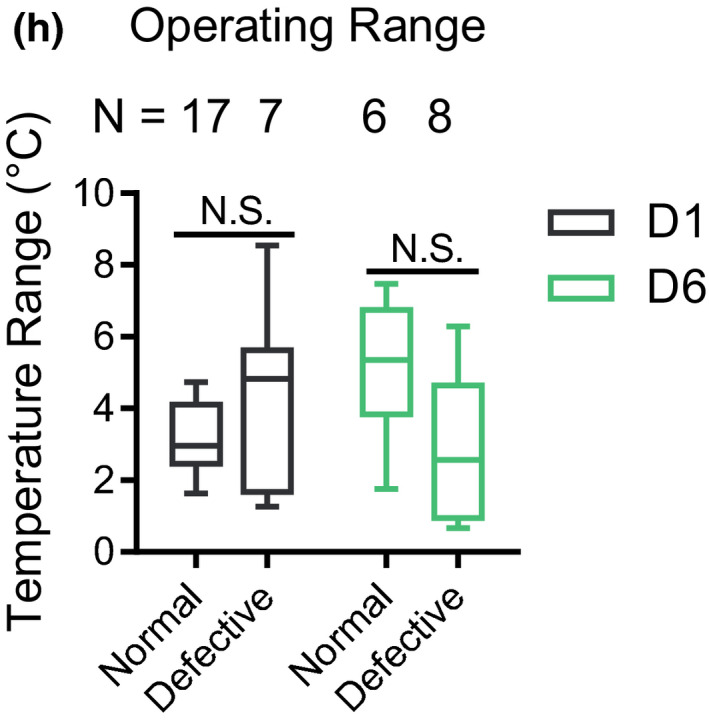

**4. In the legend of Figure 1, the statistical test:**

**Original:**

one‐way (c, e) or two‐way ANOVA (g, i) followed by *Tukey* HSD multiple comparison test.

**Correction:**

(c, e)one‐way ANOVA followed by Tukey HSD multiple comparison test.(g, i) Unpaired *t* test.

**5. In the legend of Figure S3, the statistical test:**

**Original:**

*p < 0.05, two‐way ANOVA test followed by *Tukey* HSD multiple comparison test.

**Correction:**

*p < 0.05, one‐way ANOVA test followed by *Tukey* HSD multiple comparison test.

The authors apologize for the inconvenience caused for the readers.

