# Supplementary material for: Age‐dependent changes in response property and morphology of a thermosensory neuron and thermotaxis behavior in Caenorhabditis elegans
Source: Aging Cell. 2020 Apr 19;19(5):e13146. doi: 10.1111/acel.13146 (PMC7253067; doi:10.1111/acel.13146)
Supplement: Supplementary file 1 — Supplementary Material [file ACEL-19-e13146-s001.pdf]

SUPPORTING INFORMATION

Table S1

Table S2

Supplemental Figures S1-S6

Supplemental Figure Legends

Table S1. *C. elegans* strains and transgenes used in this study

REAGENT or RESOURCE	SOURCE	IDENTIFIER
Experimental Models: Organisms/Strains		
C. elegans: Strain N2: wild isolate	CGC	WormBase: N2
C. elegans: Strain RB2464: *tax-2(ok3403)I*	CGC	WormBase: RB2464
C. elegans: Strain CLP973: *daf-16(mu86)I; oyIs18[Pgcy-8::GFP]X*	This paper	CLP973
C. elegans: *nsIs228[Psrtx-1:GFP]I*	Shai Shaham Lab	N/A
C. elegans: Strain CLP1156: *daf-2(e1370)III; oyIs18[Pgcy-8::GFP]X*	This paper	CLP1156
C. elegans: Strain VC3113: *tax-4(3771)III*	CGC	WormBase: VC3113
C. elegans: Strain FX949: *gcy-8(tm949)IV*	Mitani Lab	N/A
C. elegans: Strain IK429: *gcy-18(nj38)IV*	This paper	N/A
C. elegans Strain IK597: *gcy-23(nj37) gcy-8(oy44) gcy-18(nj38)IV*	CGC	WormBase: IK597
C. elegans: Strain PY1322: *oyIs18[Pgcy-8::GFP]X*	CGC	WormBase: PY1322
C. elegans: Strain CLP1155: *twnEx485[Pgcy-8::LifeAct:: NeonGreen(NG), gcy-8p::mCherry]*	This paper	CLP1155
C. elegans: Strain CLP1020: *twnEx435[Pgcy-8::GCaMP5G::SL2::mCherry]*	This paper	CLP1020
C. elegans: Strain IK3626: *gcy-8(tm949); njEx1565[gcy-8p::gcy-8, ges-1p::NLS::gfp]* line 1	This paper	IK3626
C. elegans: Strain IK3627: *gcy-8(tm949); njEx1565[gcy-8p::gcy-8, ges-1p::NLS::gfp]* line 2	This paper	IK3627

C. elegans: Strain IK3628: *gcy-8(tm949); njEx1565[gcy-8p::gcy-8, ges-1p::NLS::gfp]* line 3	This paper	IK3628
Recombinant DNA		
Plasmid: *Pgcy-8::GCaMP5G::SL2::mCherry*	This paper	N/A
Plasmid: *Pgcy-8::LifeAct::NeonGreen*	This paper	N/A
Plasmid: *Pgcy-8::mCherry*	This paper	N/A
Plasmid: *Pgcy-8::GFP*	This paper	N/A
Plasmid: *Pgcy-8::gcy-8*	This paper	N/A
Plasmid: *Pgcy-8::gcy-8::GFP*	This paper	N/A
Plasmid: *Pgcy-18::gcy-18::GFP*	This paper	N/A
Plasmid: *Pgcy-23::gcy-23::GFP*	This paper	N/A

Table S2. The morphology details of AFD sensory ending during aging

		% defective AFD sensory ending		
**Genotype/Age**		**Reduced or swollen mv**	**no mv and swollen center**	**Overall**
**Wild type (*oyIs18*)**				
**D1**	**N**			
Exp. 1	30	0	0	0
Exp. 2	31	19	0	19
Exp. 3	23	9	0	9
Mean		(9)	(0)	(9)
**D3**	**N**			
Exp. 1	25	24	0	24
Exp. 2	20	10	5	15
Exp. 3	22	23	5	27
Mean		(19)	(3)	(22)
**D6**	**N**			
Exp. 1	22	55	0	55
Exp. 2	27	37	4	41
Exp. 3	24	38	0	38
Mean		(43)	(1)	(44)
**D9**	**N**			
Exp. 1	18	33	17	50
Exp. 2	17	28	17	45
Exp. 3	24	54	0	54
Mean		(38)	(11)	(50)
**D12**	**N**			
Exp. 1	24	54	17	71
Exp. 2	20	45	30	75
Exp. 3	20	60	20	80
Mean		(53)	(22)	(75)
**D16**	**N**			
Exp. 1	22	82	14	95
Exp. 2	20	60	25	85

Exp. 3	17	83	11	94
Mean		(75)	(17)	(92)
**Wild type (ns*Is228*)**				
**D1**	**N**			
Exp. 1	8	0	0	0
Exp. 2	22	14	0	14
Exp. 3	20	15	0	15
Mean		(10)	(0)	(10)
**D6**	**N**			
Exp. 1	20	45	5	50
Exp. 2	13	46	0	46
Exp. 3	15	60	0	60
Mean		(50)	(2)	(52)
**D12**	**N**			
Exp. 1	17	53	0	53
Exp. 2	17	65	0	65
Exp. 3	13	54	8	62
Mean		(57)	(3)	(60)
daf-2(e1370)				
**D1**	**N**			
Exp. 1	16	19	0	19
Exp. 2	24	8	0	8
Exp. 3	23	9	0	9
Mean		(12)	(0)	(12)
**D9**	**N**			
Exp. 1	18	35	12	47
Exp. 2	21	24	10	33
Exp. 3	22	23	0	23
Mean		(27)	(11)	(34)
**D12**	**N**			
Exp. 1	25	52	0	52
Exp. 2	25	50	0	50
Exp. 3	17	59	0	59
Mean		(54)	(0)	(54)

**D16**	**N**			
Exp. 1	13	23	8	31
Exp. 2	19	42	5	47
Exp. 3	28	32	4	36
	Mean	(32)	(6)	(38)
daf-16(mu86)				
**D1**	**N**			
Exp. 1	24	17	0	17
Exp. 2	18	28	0	28
Exp. 3	24	38	0	38
	Mean	(27)	(0)	(27)
**D3**	**N**			
Exp. 1	25	52	8	60
Exp. 2	18	50	8	58
Exp. 3	23	50	8	58
	Mean	(51)	(8)	(59)
**D9**	**N**			
Exp. 1	20	70	15	85
Exp. 2	17	65	6	71
Exp. 3	21	67	0	67
	Mean	(67)	(7)	(74)
gcy-8(tm949)				
**D1**	**N**			
Exp. 1	26	4	0	4
Exp. 2	20	10	0	10
Exp. 3	28	0	0	0
	Mean	(5)	(0)	(5)
**D6**	**N**			
Exp. 1	17	12	6	18
Exp. 2	20	0	0	0
Exp. 3	19	11	0	11
	Mean	(8)	(2)	(10)
**D12**	**N**			
Exp. 1	22	27	0	27

Exp. 2	15	7	0	7
Exp. 3	12	33	0	33
Mean		(22)	(0)	(22)
gcy-8(tm949); njEx1565				
**D1**	**N**			
Exp. 1	29	42	24	66
Exp. 2	24	50	21	71
Exp. 3	23	61	9	70
Exp. 4	16	56	13	69
Mean		(52)	(17)	(69)
**D6**	**N**			
Exp. 1	16	44	6	50
Exp. 2	18	61	6	67
Exp. 3	23	74	4	78
Exp. 4	21	72	14	86
Mean		(63)	(8)	(70)
**D12**	**N**			
Exp. 1	12	58	42	100
Exp. 2	9	44	56	100
Exp. 3	9	33	56	89
Exp. 4	10	60	30	90
Mean		(49)	(46)	(95)

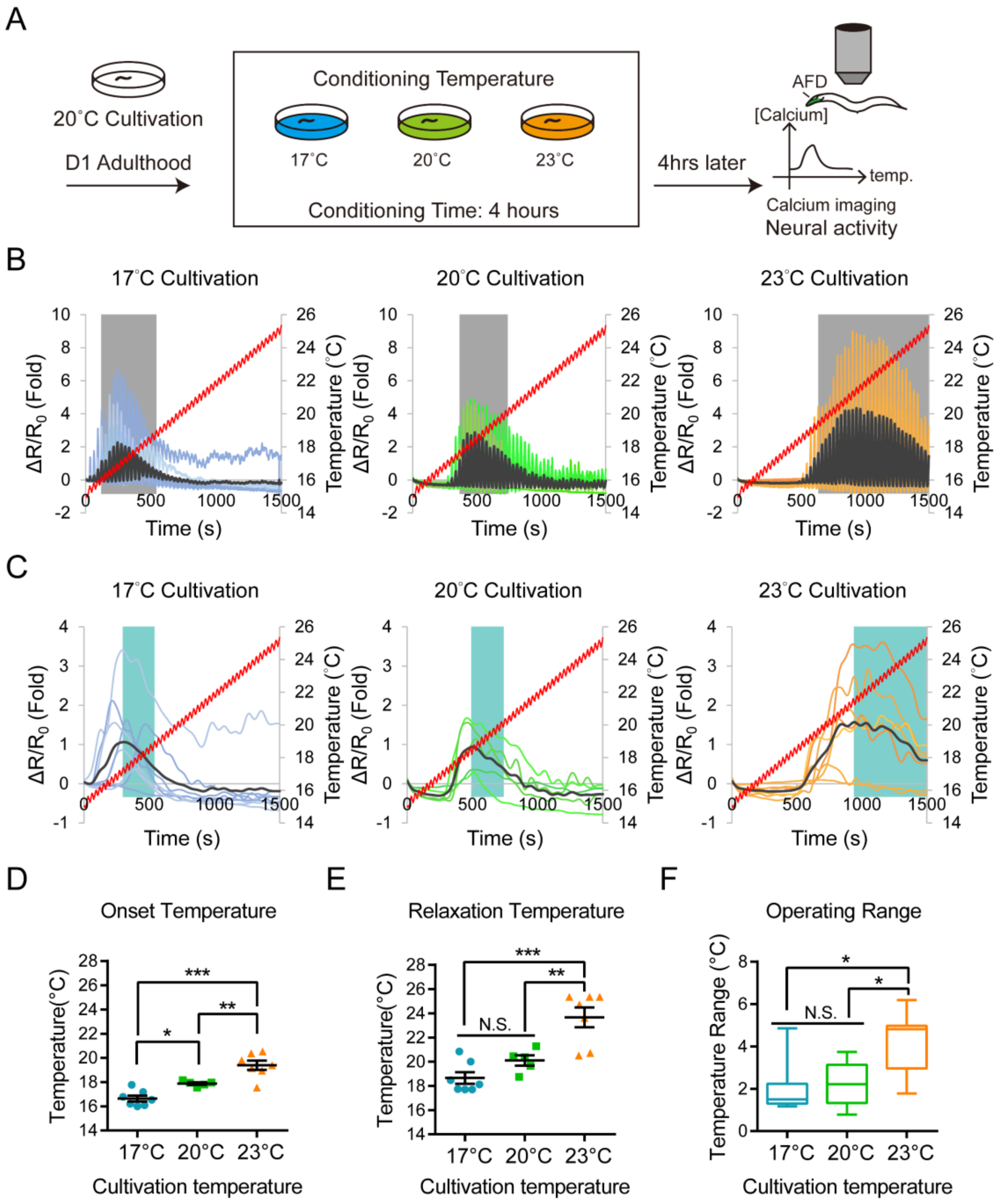

A
20˚C Cultivation
D1 Adulthood
Conditioning Temperature
17˚C
20˚C
23˚C
Conditioning Time: 4 hours
4hrs later
AFD
[Calcium]
temp.
Calcium imaging
Neural activity
B
17˚C Cultivation
20˚C Cultivation
23˚C Cultivation
ΔR/R0 (Fold)
Temperature (˚C)
Time (s)
C
17˚C Cultivation
20˚C Cultivation
23˚C Cultivation
ΔR/R0 (Fold)
Temperature (˚C)
Time (s)
D
Onset Temperature
Temperature(°C)
17°C 20°C 23°C
Cultivation temperature
E
Relaxation Temperature
Temperature(°C)
N.S.
17°C 20°C 23°C
Cultivation temperature
F
Operating Range
Temperature Range (°C)
N.S.
17°C 20°C 23°C
Cultivation temperature

Figure S1. Experience-dependent response property of AFD is recapitulated with the oscillating warming paradigm. **(A)** Schematic diagram of the experimental workflow. To avoid the influence of temperature on aging or development, we culture animals at 20°C and shift the cultivation temperature 4 hours before calcium imaging. **(B)** The calcium dynamics of AFD, y(t), in animals cultivated at 17°C, 20°C and 23°C, respectively, with distinct onset temperature. Colored traces represent individual recordings and the black traces are group average. Red curves are temperature stimulation. Gray shades mark the average operating range of AFD calcium dynamics recorded from the soma. **(C)** The slow calcium dynamics of AFD, $y^{sl}(t)$. Colored traces are $y^{sl}(t)$ in individual recordings and black traces represent group average. Blue shades mark the average relaxation phase of $y^{sl}(t)$. **(D-F)** The onset temperature **(D)**, relaxation temperature **(E)** and operating range **(F)** of AFD calcium dynamics. N = 5-7 animals in each cultivation condition. Error bars = S.E.M. *, $p < 0.05$, **, $p < 0.01$, ***, $p < 0.001$, N.S., not significant, one-way ANOVA test followed by *Tukey* HSD multiple comparison test.

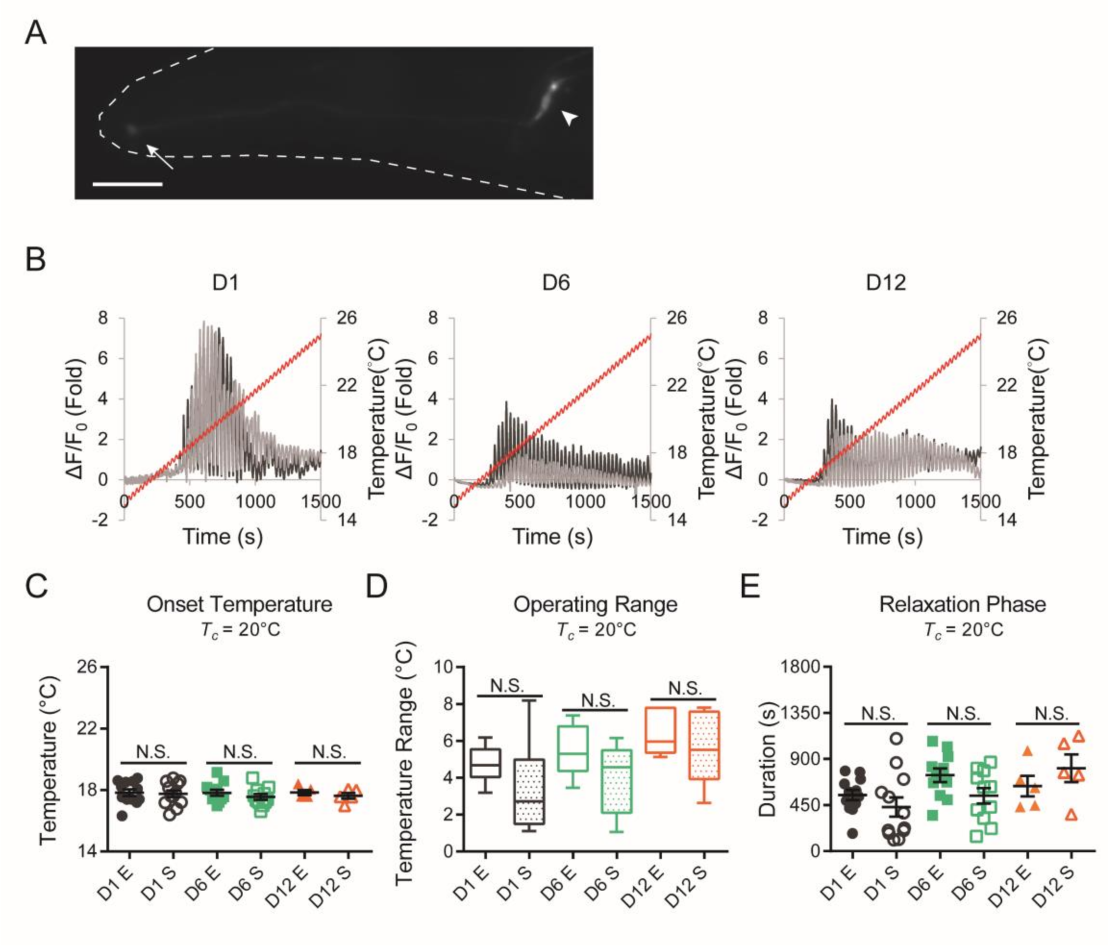

Figure S2. Simultaneous recording of calcium dynamics from the sensory ending and soma of AFD in animals cultivated at 20°C. **(A)** A snapshot of simultaneous calcium imaging of the AFD sensory ending and soma in the same frame. The dashed line indicates the profile of the adult worm head. Arrow, the sensory ending; arrowhead, the soma. Scale bar = 10 μm. **(B)** Superimposed temperature-evoked calcium dynamics recorded simultaneously from the sensory ending (light gray) and the soma (dark gray) in individual animals at indicated age. the red line indicates temperature stimulation as described in Figure 3A. **(C-E)** Quantification of temperature at AFD activation onset **(C)**, AFD operating range **(D)**and AFD relaxation phase **(E)** recorded from sensory ending and soma of the same neuron. N = 12, 11 and 5 neurons recorded in D1, D6 and D12 animals, respectively.

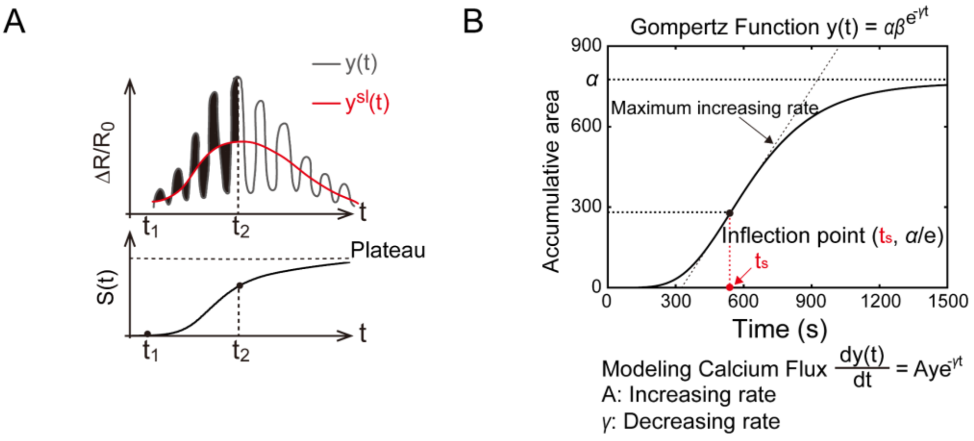

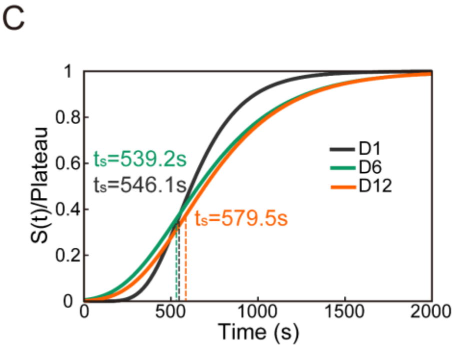

D

Age	Final set of parameters	Asymptotic Standard Error
D1	α = 0.972739	± 0.0003563 (0.03663%)
	β = 6.6791 x 10^{-8}	± 4.669 x 10^{-9} (6.99%)
	γ = 0.00513623	± 7.712 x 10^{-6} (0.1501%)
D6	α = 1.03628	± 0.0005123 (0.04944%)
	β = 0.00550421	± 6.142 x 10^{-5} (1.116%)
	γ = 0.00305853	± 4.361 x 10^{-6} (0.1426%)
D12	α = 1.01743	± 0.001031 (0.1013%)
	β = 0.00197843	± 4.931 x 10^{-5} (2.492%)
	γ = 0.00315543	± 8.077 x 10^{-6} (0.256%)

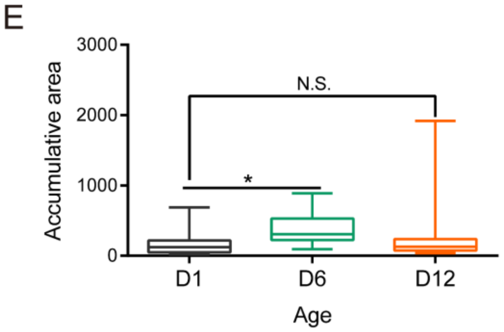

Figure S3. Quantitative Analysis and Modeling of AFD calcium flux of animals cultivated at 20°C. **(A)** Schematic diagram of the cumulative AFD calcium flux, S(t), in a time series. **(B)** Schematic diagram of the parameters used for fitting of S(t) by Gompertz function. **(C)** Switching time t_s for AFD calcium response in Figure 3B. N = 27, 23 and 12 neurons recorded in D1, D6 and D12 animals, respectively. **(D)** The parameters of S(t) for fitting the experimental results of the averaged D1, D6 and D12 AFD accumulative calcium flux. **(E)** Accumulative area between y(t) and $y^{sl}(t)$ of AFD responses at the high temperature range (21°C-25°C). N = 27, 23 and 12 neurons recorded in D1, D6 and D12 animals used in Figure 3B, respectively. Error bars = S.E.M. N.S., not significant, *, $p < 0.05$, two-way ANOVA test followed by *Tukey* HSD multiple comparison test.

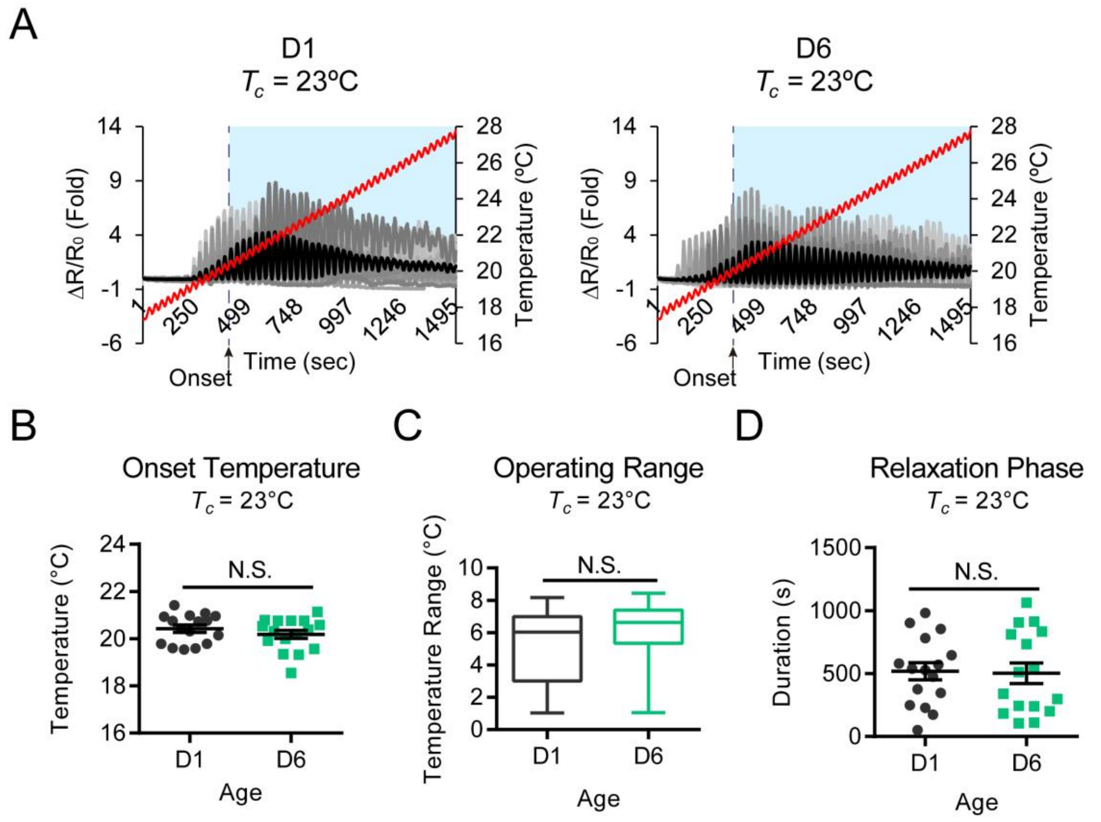

Figure S4. AFD Response Property Are Similar in D1 and D6 Animals Cultivated at 23°C. **(A, B)** The calcium dynamics of AFD in D1 (A) and D6 (B) animals cultivated at 23°C, respectively. Colored traces represent individual recordings and the black traces are group average. Red curves are temperature stimulation. Blue shades mark the average operating range of AFD calcium dynamics recorded from the soma. **(C-E)** The onset temperature **(D)**, operating range **(E)** and relaxation phase **(F)** of AFD calcium dynamics. N= 16 neurons recorded in each group. Error bars = S.E.M. N.S., not significant, Unpaired *t*-test.

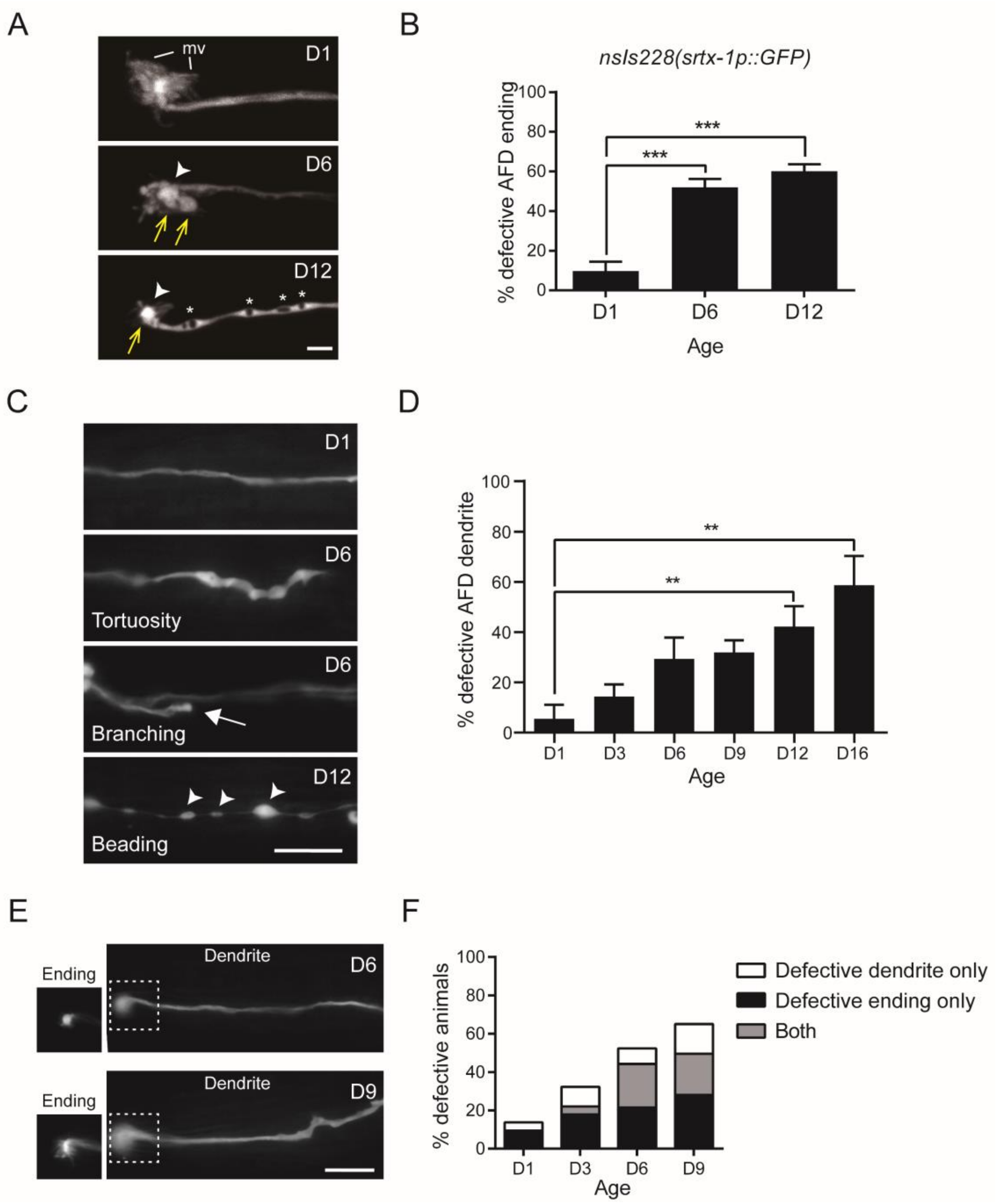
A
mv
D1
D6
D12
B
nsIs228(srtx-1p::GFP)
% defective AFD ending
***
***
D1
D6
D12
Age
C
D1
D6
Tortuosity
D6
Branching
D12
Beading
D
% defective AFD dendrite
**
**
D1
D3
D6
D9
D12
D16
Age
E
Ending
Dendrite
D6
Ending
Dendrite
D9
F
% defective animals
Defective dendrite only
Defective ending only
Both
D1
D3
D6
D9
Age

Figure S5. Age-dependent changes in AFD sensory endings and dendrites. **(A)** Fluorescent confocal images of the AFD sensory ending during aging. AFD is marked by the transgene *nsIs228(srtx-1p::GFP)*. Arrows indicate microvilli and arrowheads indicate engorged structures in AFD sensory ending. Asterisks indicate bubble-1ike lesions along the dendrite. Scale bar = 2 µm. **(B)** Quantification of age-dependent changes in AFD sensory endings marked by *nsIs228(srtx-1p::GFP)*. N = 3 independent experiments with 8-22 animals for each experiment at each time points. Total numbers of AFD neurons scored: D1 = 50, D6 = 48, D12 = 47. Error bars = S.E.M. ***, $p < 0.001$, one-way ANOVA test followed by *Tukey* HSD multiple comparison test. **(C, D)** Epifluorescent images **(C)** and quantification **(D)** of AFD dendrite defects marked by *oyIs18(gcy-8p::GFP)* at indicated age. Scale bar = 10 µm. Error bars = S.E.M. **, $p < 0.01$, one-way ANOVA test followed by *Tukey* HSD multiple comparison test. **(E)** Epifluorescent images of dendrite and sensory ending defects from two individual animals at indicated age. The upper panels show a D6 AFD neuron with defective sensory ending and an intact dendrite. The lower panels show a D9 AFD neuron with normal sensory ending but a tortuous dendrite. Scale bar = 10 µm. **(F)** Quantification of age-dependent AFD defects grouped by the presence of changes in the dendrite, sensory ending, or both. Animals analyzed are from the three independent cohorts previously scored in Figure 4C. N = 59-84 analyzed neurons in each age cohort.

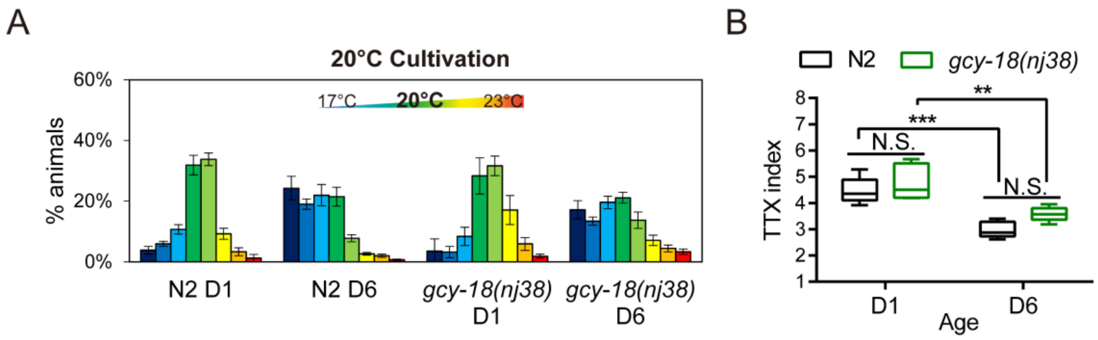

Figure S6. Thermotaxis behaviors of young and aged *gcy-18(nj38)* animals cultivated at 20°C. **(A, B)** The distribution **(A)** and TTX index **(B)** of D1 and D6 wild-type and *gcy-18(nj38)* animals on a 17°C-23°C temperature gradient. N = 6-7 assays. Animals are maintained at 20°C prior to the TTX assay. Boxes represent mean ± the first and the third quartile. **, $p < 0.01$, ***, $p < 0.001$, N.S., not significant, two-way ANOVA followed by *Tukey* HSD multiple comparison test.
